# AKT controls protein synthesis and oxidative metabolism via combined mTORC1 and FOXO1 signalling to govern muscle physiology

**DOI:** 10.1002/jcsm.12846

**Published:** 2021-11-09

**Authors:** Natasha Jaiswal, Matthew Gavin, Emanuele Loro, Jaimarie Sostre‐Colón, Paul A. Roberson, Kahealani Uehara, Nicole Rivera‐Fuentes, Michael Neinast, Zoltan Arany, Scot R. Kimball, Tejvir S. Khurana, Paul M. Titchenell

**Affiliations:** ^1^ Institute for Diabetes, Obesity, and Metabolism Perelman School of Medicine at the University of Pennsylvania Philadelphia PA USA; ^2^ Department of Physiology Perelman School of Medicine at the University of Pennsylvania Philadelphia PA USA; ^3^ Penn Muscle Institute, Department of Physiology Perelman School of Medicine at the University of Pennsylvania Philadelphia PA USA; ^4^ Department of Cellular and Molecular Physiology Penn State College of Medicine Hershey PA USA; ^5^ Cardiovascular Institute Perelman School of Medicine at the University of Pennsylvania Philadelphia PA USA

**Keywords:** AKT signalling, Disuse‐induced muscle wasting, Fibre specification, Insulin action

## Abstract

**Background:**

Skeletomuscular diseases result in significant muscle loss and decreased performance, paralleled by a loss in mitochondrial and oxidative capacity. Insulin and insulin‐like growth factor‐1 (IGF‐1) are two potent anabolic hormones that activate a host of signalling intermediates including the serine/threonine kinase AKT to influence skeletal muscle physiology. Defective AKT signalling is associated with muscle pathology, including cachexia, sarcopenia, and disuse; however, the mechanistic underpinnings remain unresolved.

**Methods:**

To elucidate the role of AKT signalling in muscle mass and physiology, we generated both congenital and inducible mouse models of skeletal muscle‐specific AKT deficiency. To understand the downstream mechanisms mediating AKT's effects on muscle biology, we generated mice lacking AKT1/2 and FOXO1 (M‐AKTFOXO1TKO and M‐indAKTFOXO1TKO) to inhibit downstream FOXO1 signalling, AKT1/2 and TSC1 (M‐AKTTSCTKO and M‐indAKTTSCTKO) to activate mTORC1, and AKT1/2, FOXO1, and TSC1 (M‐QKO and M‐indQKO) to simultaneously activate mTORC1 and inhibit FOXO1 in AKT‐deficient skeletal muscle. Muscle proteostasis and physiology were assessed using multiple assays including metabolic labelling, mitochondrial function, fibre typing, *ex vivo* physiology, and exercise performance.

**Results:**

Here, we show that genetic ablation of skeletal muscle AKT signalling resulted in decreased muscle mass and a loss of oxidative metabolism and muscle performance. Specifically, deletion of muscle AKT activity during development or in adult mice resulted in a significant reduction in muscle growth by 30–40% (*P*  < 0.0001; *n* = 12–20) and 15% (*P* < 0.01 and *P* < 0.0001; *n* = 20–30), respectively. Interestingly, this reduction in muscle mass was primarily due to an ~40% reduction in protein synthesis in both M‐AKTDKO and M‐indAKTDKO muscles (*P* < 0.05 and *P* < 0.01; *n* = 12–20) without significant changes in proteolysis or autophagy. Moreover, a significant reduction in oxidative capacity was observed in both M‐AKTDKO (*P* < 0.05, *P* < 0.01 and *P* < 0.001; *n* = 5–12) and M‐indAKTDKO (*P* < 0.05 and *P* < 0.01; *n* = 4). Mechanistically, activation and inhibition of mTORC1/FOXO1, respectively, but neither alone, were sufficient to restore protein synthesis, muscle oxidative capacity, and muscle function in the absence of AKT *in vivo*. In a mouse model of disuse‐induced muscle loss, simultaneous activation of mTORC1 and inhibition of FOXO1 preserved muscle mass following immobilization (~5–10% reduction in casted M‐indFOXO1TSCDKO muscles vs. ~30–40% casted M‐indControl muscles, *P* < 0.05 and *P* < 0.0001; *n* = 8–16).

**Conclusions:**

Collectively, this study provides novel insights into the AKT‐dependent mechanisms that underlie muscle protein homeostasis, function, and metabolism in both normal physiology and disuse‐induced muscle wasting.

## Introduction

Skeletal muscle is composed of highly specialized, heterogeneous fibres that quickly adapt to changes in the hormonal and physical environment. This plasticity of muscle is a reflection of variations in the structure and function of muscle fibres defined in part by their fibre‐type composition. Musculoskeletal diseases are associated with a significant reduction in muscle size and alterations in fibre composition and strength, leading to an increase in morbidity and mortality.^1^ These myopathies stem from a diverse range of insults such as inactivity (immobilization, ageing, or nerve injury), cancer cachexia, or diabetes. Therefore, a better mechanistic understanding of the signals that integrate muscle growth, fibre‐type specification, and function in physiological and skeletomuscular diseases is of utmost importance for the development of new therapeutics.

Insulin and insulin‐like growth factor‐1 (IGF‐1) are potent anabolic hormones that influence the systemic regulation of protein, lipid, and glucose metabolism. In particular, insulin and IGF‐1 are powerful inducers of muscle mass and are critical for muscle glucose homeostasis. As a result, there is considerable interest in delineating the key downstream signalling mechanisms responsible for mediating insulin/IGF‐1's anabolic effects on muscle. Earlier studies implicate the serine/threonine kinase AKT (protein kinase B) as an important insulin/IGF‐1‐dependent signalling node in muscle.^2^ Over the last several decades, rigorous cell culture experiments, coupled with overexpression genetic studies in animal models, support the concept that AKT is a key intermediate in insulin/IGF‐1‐mediated muscle hypertrophy.^3^, ^4^ Consistent with this notion, AKT activity is reduced in several models of muscle wasting including diabetes, sarcopenia, and immobilization.^5^, ^6^, ^7^ Collectively, these studies identified AKT as an important regulator of skeletal muscle mass and suggest that defective AKT signalling may underlie some skeletomuscular diseases. Therefore, understanding the AKT‐dependent mechanisms governing skeletal muscle physiology is critical to our understanding of both physiology and pathophysiology.

The coordinated balance of proteostasis in concert with fibre specification is critical for muscle function and performance. Understanding the molecular mediators of fibre‐type specification and connection to hormonal and environmental changes is of significant importance to our understanding of skeletal muscle biology. Previous studies show that the mammalian target of rapamycin complex 1 (mTORC1), a key regulatory protein involved in cell growth, acts downstream of AKT to promote muscle hypertrophy by insulin/IGF‐1.^3^, ^4^ On the other hand, *in vitro* studies suggest an mTORC1‐independent role of insulin/IGF‐1 in the inhibition of protein degradation via inhibition of forkhead box O (FOXO) transcription factors and reduction in muscle atrogenes, Murf‐1 and Atrogin‐1.^8^, ^9^ While these seminal studies are critical to our understanding of the specific AKT‐dependent pathways involved in muscle growth, recent attempts to establish these mechanisms *in vivo* question the sufficiency of these pathways alone. For example, knockout of the insulin receptor (IR) and IGF‐1 receptor (IGF‐1R) in muscles causes a switch towards an oxidative phenotype and induces muscle loss via FOXO‐mediated proteolysis. Paradoxically, these muscles display an uncharacterized up‐regulation in basal AKT and mTORC1 activity despite no intact insulin or IGF‐1 signalling.^8^ Conversely, mice with genetic ablation of mTORC1 in muscle (RAMKO) exhibit features of slow/oxidative fibres and exhibit muscle loss despite activation of AKT and reduction in FOXO activity due to a classic negative feedback loop.^10^ Moreover, sustained activation of mTORC1 is not sufficient to induce muscle growth and increase oxidative capacity in all muscles potentially due to reduced AKT activity following negative feedback inhibition by mTORC1.^11^ Thus, while the role of AKT in inducing muscle hypertrophy is well established, there remains controversy over the specific AKT‐dependent downstream mediators of muscle growth and function *in vivo*. In addition, much less is known about the role of AKT and its distal signals in the regulation of skeletal muscle fibre‐type composition, metabolic function, and muscle performance.

In this manuscript, we sought to establish the role of AKT signalling in controlling muscle fibre size, fibre‐type specification, and function *in vivo*. Moreover, we aimed to identify the specific signalling mechanisms underlying muscle wasting in response to immobilization. Here, we provide evidence that muscle AKT is required for the maintenance of the slow‐oxidative fibre type and protein synthesis (without altering rates of protein degradation) to control muscle fibre size and function. We observed that this effect requires simultaneous integration of both FOXO1 mTORC1 pathways, rather than these pathways working in parallel, to control fibre specification and function downstream of AKT. Lastly, we demonstrate that mimicking distal AKT signalling, by combined genetic activation of mTORC1 and inhibition of FOXO1, preserves muscle mass in response to immobilization‐induced muscle wasting.

## Methods

### Mice

Male mice in the age group of 8–12 weeks were used in all the experiments. M‐AKTDKO mice were generated by crossing *AKT1*_*loxp/loxp*_ *;AKT2*_*loxp/loxp*_ floxed mice with mice carrying the Cre recombinase driven by a skeletal muscle actin promoter, ACTA1–Cre [human skeletal actin (HSA)–CRE] (Jackson Laboratory, Stock Number 006149) as previously described.^12^ For muscle‐specific deletion of AKT1/2–FOXO1, AKT1/2–TSC1, and AKT1/2–FOXO1–TSC1, *AKT1*_*loxp/loxp*_ *;AKT2*_*loxp/loxp*_ *;FOXO1*_*loxp/loxp*_, *AKT1*_*loxp/loxp*_ *;AKT2*_*loxp/loxp*_ *;TSC1*_*loxp/loxp*_, and *AKT1*_*loxp/loxp*_ *;AKT2*_*loxp/loxp*_ *;FOXO1*_*loxp/loxp*_ *;TSC1*_*loxp/loxp*_ mice described here were crossed with transgenic mice harbouring HSA–CRE promoter. Littermates lacking the HSA transgene (Cre‐) served as controls and were pooled together. Alternatively, *AKT1*_*loxp/loxp*_ *;AKT2*_*loxp/loxp*_, *AKT1*_*loxp/loxp*_ *;AKT2*_*loxp/loxp*_ *;FOXO1*_*loxp/loxp*_, *AKT1*_*loxp/loxp*_ *;AKT2*_*loxp/loxp*_ *;TSC1*_*loxp/loxp*_, *AKT1*_*loxp/loxp*_ *;AKT2*_*loxp/loxp*_ *;FOXO1*_*loxp/loxp*_ *;TSC1*_*loxp/loxp*_, and *FOXO1*_*loxp/loxp*_ *;TSC1*_*loxp/lox*_ floxed mouse lines were crossed to mice containing the Cre recombinase–oestrogen receptor fusion protein under the control of human ACTA‐1 (HSA–ESR–CRE) (Jackson Laboratory, Stock Number 031934), allowing tamoxifen‐inducible Cre‐mediated recombination in adult muscle. Floxed mice (8–12 weeks of age) for the indicated genotype that lack the HSA–ESR–CRE transgene (M‐indControl) or mice positive for the transgene (indKOs) were injected with tamoxifen IP (100 mg/kg) for five consecutive days to induce knockout. Experiments were performed 4–7 weeks after tamoxifen. All animal experiments were reviewed and approved by the University of Pennsylvania IACUC in accordance with the NIH guidelines.

### Western blotting

Protein lysates were prepared as described previously.^12^ The antibodies used are as follows: FOXO1 (#2880 CST), AKT2 (#2964 CST), HSP90 (#4874 CST), ribosomal protein S6 (#2217 CST), phospho‐S6 ribosomal protein (Ser240/244) (#5364 CST), AKT (pan) (#4691 CST), TSC1 (#6935), TOM20 (#42406 CST), FOXO3a (#12829S), phospho‐PRAS40 (Thr246) (#2997S), phospho‐AKT2 (Ser474) (#8599S), phospo‐AKT1/2 (Ser473) (#4060), phospho‐FOXO1 (Ser256) (#9461L), FOXO1 (L27) (#9454S), FOXO1 (C29H4) (#2880S), p62 (#5114S), phospho‐4E‐BP1 (#2855S), 4E‐BP1 (#9452S), and LC3A/B (#4108) from Cell Signaling, OXPHOS rodent antibody (#ab110413) from Abcam, puromycin (#EQ 0001) from Kerafast, donkey anti‐rabbit (#926‐32213) from LI‐COR, and mouse IgG heavy and light chain HRP‐conjugated antibody (#A90‐116P) from Bethyl Laboratories.

### Histology and immunohistochemistry

Muscles isolated were embedded in optimal cutting temperature compound (OCT compound) prior to freezing in liquid nitrogen. Samples were cut into 10 μm cross sections. General histology on cross sections was performed using hematoxylin (Fisher Scientific) and eosin (Sigma) stains. For fibre‐type staining (immunostaining), cross sections were blocked with 1% BSA/PBS for 30 min and incubated for 1 h at room temperature with specific primary antibody against myosin heavy chain (MHC) type I (BA‐F8), MHC type IIa (SC‐71), and MHC type IIb (BF‐F3) from Developmental Studies Hybridoma Bank. Unstained fibres (black) were defined as MHC type IIx. Samples were subsequently washed with 1% BSA/PBS, three times for 1 h, and stained with appropriate fluorescently labelled secondary antibodies for 1 h at room temperature. After washing with PBS, samples were mounted with ProLong (Thermo Fisher Scientific) and imaged using Nikon E600 microscope.

### Gene expression analysis

Total RNA was isolated from the frozen muscles using the RNeasy Plus Kit from Qiagen, and complementary DNA was synthesized using Moloney murine leukaemia virus reverse transcriptase as previously described.^12^ Relative expression of the genes of interest was quantified by real‐time PCR using the SYBR Green dye‐based assay.

### Physiological measurements

Forced treadmill exercise tests were performed by running 8‐ to 12‐week‐old mice on a treadmill equipped with an electrical shock grid (Columbus Instruments, Columbus, OH). Mice were acclimated to the treadmill for 20 min on two consecutive days without starting of the treadmill. For the test, mice were subjected to runs starting at a velocity of 10 m/min and increased by 5 m/min every 5 min until exhaustion. Total distance run and time to exhaustion were calculated. Spontaneous wheel running (Columbus Instruments) was monitored for approximately 7 days during which mice were single housed in cages with *ad libitum* access to food and water. Wheel cage revolutions were monitored every day, and the accumulated running distance was calculated at the end of 5 days. The muscle strength in the forelimbs was measured with a grip meter (Vernier LabPro). The mice were trained to grasp a horizontal mesh while being pulled by their tail, and the force was detected by a sensor. Investigator was blinded during the experiment protocol. Briefly, mice were trained to grasp a horizontal metal bar while being pulled by their tail and the force was detected by a sensor. Ten measurements were determined for each mouse and averaged. Mouse locomotor activity of 8‐ to 12‐week‐old male mice was measured using Comprehensive Laboratory Animal Monitoring System (Columbus Instruments) metabolic cages housed within environment‐controlled rodent incubators.

### *Ex vivo* physiology

Muscle physiological analysis was performed on isolated extensor digitorum longus (EDL) and soleus muscles using a 1200A Intact Muscle Test System equipped with Dynamic Muscle Control v.5.415 software (Aurora Scientific, Aurora, ON, Canada) at the Muscle Physiology Assessment Core of the Pennsylvania Muscle Institute. Muscles were dissected and analysed in constantly oxygenated Ringer's solution (100 mM NaCl, 4.7 mM KCl, 3.4 mM CaCl_2_, 1.2 mM KH_2_PO_4_, 1.2 mM MgSO_4_, 25 mM HEPES, and 5.5 mM d‐glucose) at 24°C. Maximal isometric twitch and tetanic contractions were obtained by direct electrical field stimulation propagated from two plate electrodes using a single 0.2 ms pulse or 120 Hz train lasting 500 ms, respectively. Five minutes was allowed between tests to ensure muscle recovery. Specific force was determined by normalizing absolute force to muscle cross‐sectional area (CSA).^13^ CSA was calculated by dividing the muscle mass by the product of the muscle density coefficient (1.06 g/cm^3^), muscle optimal length (Lo), and the fibre length coefficient (0.45 for EDL and 0.69 for soleus).^13^

### Proteolysis assay

Proteolysis was measured in isolated soleus and EDL. Muscles were preincubated in 1 mL of KRB buffer (in mM: 117 NaCl, 4.7 KCl, 2.5 CaCl_2_, 1.2 KH_2_PO_4_, 1.2 MgSO_4_, 24.6 NaHCO_3_, and 5 glucose) for 30 min with constant bubbling of 95% O_2_/5% CO_2_ before transferring to fresh 1 mL of KRB containing 0.5 mM cycloheximide to inhibit protein synthesis for 2 h. Incubation buffer was collected, and tyrosine concentration was determined in 0.5 mL of incubation buffer by measuring the fluorescence at 436 nm excitation and 535 emission wavelength and normalized to muscle weight.

### Proteasome activity assays

Proteasome activity was determined from muscle homogenates using substrates for peptidylglutamyl‐like activity of the 26S proteasome as previously described^8^ using Z‐Leu‐Leu‐Glu‐7‐amido‐4‐methylcoumarin (LLE) (C0483; Sigma‐Aldrich; 100 μM final concentration) as the substrate. Proteasome activity was measured by the amount of fluorophore cleaved from the substrate by the proteasome and compared against a standard curve of 7‐amido‐4‐methylcoumarin (A9891; Sigma‐Aldrich).

### Protein fractional synthesis rate

The rate of protein synthesis was measured by the incorporation of ^2^H_2_O (4% in drinking water) in mice over 36 h. Mice were harvested in either fasted condition (16 h) or refed for 1 h (following 16 h fasting) with normal chow. Blood was collected via cardiac puncture, and skeletal muscle (gastrocnemius) was dissected and snap‐frozen in liquid nitrogen, and plasma was isolated and frozen. Muscle protein turnover was measured in the tissues that were homogenized and delipidated with chloroform:methanol (2:1). Following centrifugation to separate phases, the protein layer was washed with 10% and then 5% TCA and subjected to hydrolysis overnight with 6 N HCl at 110°C. The muscle protein hydrolysate was run over AG50W‐X8 resin and washed with distilled water and amino acids eluted with 3 M NH_4_OH. The eluate was dried under nitrogen and the amino acids converted to their heptafluorobutyryl isobutyl ester derivatives. Derivatized samples were injected into an Agilent 7890A/5975C series gas chromatograph/mass spectrometer (GC/MS) fitted with an Agilent DB5‐MS column operated under methane negative chemical ionization. The unlabelled and [^2^H]‐labelled alanine derivatives were monitored at *m*/*z* 321–323 to determine alanine enrichment with deuterium. Plasma D_2_O enrichment was determined GC/MS following deuterium exchange with acetone as previously described.^14^

### Transmission electron microscopy

For electron microscopy, tissues for electron microscopic examination were fixed with 2.5% glutaraldehyde and 2.0% paraformaldehyde in 0.1 M sodium cacodylate buffer, pH 7.4, overnight at 4°C. After subsequent buffer washes, the samples were post‐fixed in 2.0% osmium tetroxide with 1.5% K_3_Fe(CN)_6_ for 1 h at room temperature and rinsed in DH_2_O. After dehydration through a graded ethanol series, the tissues were infiltrated and embedded in EMbed 812 (Electron Microscopy Sciences, Fort Washington, PA). Thin sections were stained with uranyl acetate and SATO lead and examined with a JEOL 1010 electron microscope fitted with a Hamamatsu digital camera and AMT Advantage NanoSprint500 software.

### Casting experiment for disuse‐induced muscle wasting

One of the hindlimbs was immobilized as described previously.^15^ Mice were anaesthetised using isoflurane, both the hindlimbs were shaved using hair clippers, and the skin was swabbed with 70% alcohol. One of the hindlimbs was wrapped with a single layer of surgical tape, and a 2 mL Eppendorf tube was fastened to the tape using a super glue to allow the foot to be held in the neutral position. The bottom of the tube was also removed to prevent condensation inside the tube. The weight of the tube was ~500 mg (~2% of body weight) and did not appear to limit animal mobility. The animals were left with the casts for 7 days, following which muscles were harvested and the difference in the muscle mass from the non‐immobilized contralateral leg was recorded.

### Metabolomics

Muscle amino acid levels were quantified by the Metabolomics Core of the Diabetes Research Center at the University of Pennsylvania as previously described^12^ in the gastrocnemius muscles in *ad libitum* state. Data analyses were performed using Maven software, which allowed for sample alignment, feature extraction, and peak picking. Extracted ion chromatogram for each metabolite was manually examined to obtain its signal, using a custom‐made metabolite library.

### Puromycin incorporation for protein synthesis

Skeletal muscle protein synthesis was measured by the incorporation of puromycin into peptide chains as described previously.^15^ Briefly, mice were fasted overnight and refed for 1 h on the day of harvest followed by intraperitoneal injection of puromycin dihydrochloride (AG Scientific, San Diego, CA) in saline (0.040 μmol puromycin/g body weight) via. Thirty minutes after injection, the gastrocnemius muscle was removed and snap‐frozen in liquid nitrogen before being subjected to immunoblot analysis using an anti‐puromycin antibody. Quantification of the blot was performed by normalizing the intensity of the puromycinylated proteins with the combined intensity of the IgG heavy and light chain as previously described.^15^

### Serum metabolite extraction and derivatization for Gas Chromatography–Mass Spectrometry

Serum samples and a six‐sample calibration set of amino acids were spiked with a heavy isotope of leucine as an internal standard and then extracted in HCl and methanol, dried, and then silylated in acetonitrile. Specifically, 10 μL of serum or standard amino acids was added to 16.7 μL of water, and this mixture was spiked with 8 μL of 1.2 mM U13C‐15N‐leucine (Cambridge Isotope CNLM‐281‐H). This mixture was added to 2.4 μL of 0.4 N HCl and 148 μL of 100% methanol, vortexed, and incubated on dry ice for 15 min. Samples were then centrifuged for 15 min at 16 000 *g* and 4°C, and equal volumes of supernatant were collected and dried in a SpeedVac at room temperature. Dried metabolites were derivatized at 70°C for 90 min in 40 μL of a 1:1 mixture of acetonitrile (Sigma 34851) and *N*‐*tert*‐butyldimethylsilyl‐*N*‐methyl trifluoroacetamide (MTBSTFA) (Sigma 394882). After derivatization, samples were cooled to room temperature and centrifuged for 5 min at 10 000 *g*, and the supernatant was transferred to vials for GC–MS analysis.

### Serum metabolite measurement by Gas Chromatography–Mass Spectrometry

One microlitre of the sample was injected via automatic liquid sampler (Agilent 7693A) into an Agilent 7890B GC coupled with an Agilent 5977B mass selective detector (MSD) (Agilent Technologies). The GC was operated in splitless injection mode with helium as the carrier gas at a flow rate of 1.2 mL/min. The GC column was a 30 m × 250 μm × 0.25 μm HP‐5ms Ultra Inert column (Agilent 19091S‐433UI). The inlet temperature was 250°C, and, after 3 min at 100°C, the oven temperature program was increased as follows: 4°C/min to 230°C then 20°C/min to 300°C and hold 5 min. The transfer line temperature was 250°C, and the MSD source and quadrupole temperatures were 230°C and 150°C, respectively. After a 6 min solvent delay, the MSD was operated in electron ionization mode and scan mode with a mass range of 50–550 AMU at 2.9 scans/s. Agilent Mass Hunter Quantitative Analysis software was used for quantification of chromatograms. The absolute concentration of each amino acid was determined by fitting the ion counts normalized to internal standard onto a calibration curve constructed from the set of calibration samples prepared and run in parallel.

### Mitochondrial respiration

Real‐time mitochondrial oxygen consumption rates were measured in the isolated mitochondria (150 μg) from gastrocnemius muscles as described previously^12^ in the presence of pyruvate/malate for State 2 respiration, ADP for State 3 respiration, or oligomycin for State 3 respiration.

### Statistical analysis

All data are presented as means ± SEM. The statistical analysis with one‐way ANOVA was performed when more than two genotypes with one condition were compared followed by Tukey's multiple comparisons test, with two‐way ANOVA when multiple genotypes and two conditions were involved followed by Tukey's multiple comparisons test, and with Student's *t*‐test when only two groups of data were compared. *P* < 0.05 was deemed to be significant.

## Results

### Inhibition of AKT reduces muscle mass via inhibition of protein synthesis without altering rates of muscle proteolysis

Earlier studies have demonstrated that muscle‐specific overexpression of an activated form of AKT1 was sufficient to stimulate skeletal muscle hypertrophy,^4^, ^16^ implicating an important role for AKT in controlling muscle mass *in vivo*. AKT exists in three isoforms in mammals AKT 1–3 (also known as PKB α/β/γ). AKT1 is ubiquitously expressed (accounting for <10% of muscle AKT), AKT2 is selectively expressed in metabolic tissues including skeletal muscle (contributing to ~90% of total AKT in muscle), and AKT3 is enriched in brain and testes.^12^, ^17^ Consistent with data showing that AKT is sufficient to drive muscle hypertrophy, we have previously reported that deletion of both AKT isoforms expressed in muscle AKT1 and AKT2, but neither alone, resulted in significant loss in muscle mass indicating that AKT is both required and sufficient for muscle growth *in vivo*.^12^

**Figure 1:**
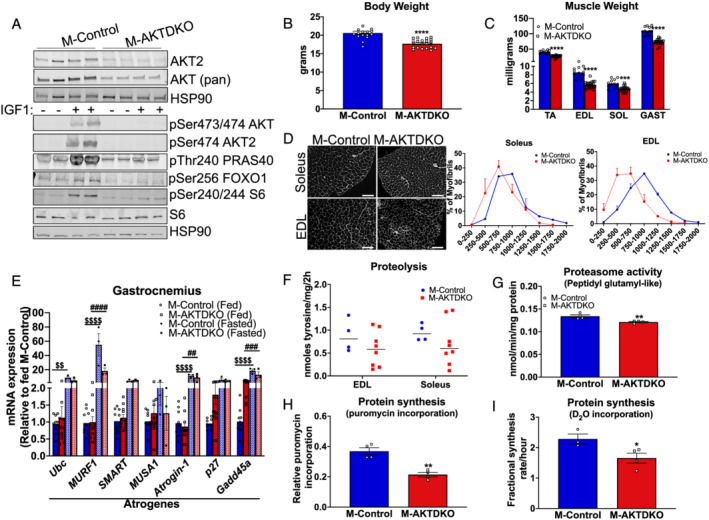
Congenital deletion of skeletal muscle AKT reduces muscle mass via reduced protein synthesis without altering protein degradation. (A) Western blots for AKT2, AKT (pan), HSP90 protein, phospho‐AKT, phospho‐S6, S6, phospho‐FOXO1, and HSP90 in gastrocnemius from M‐AKTDKO and control mice treated with IGF‐1 for 1 h following 16 h fasting. (B) Body weight of 8‐ to 12‐week‐old mice (*n* = 16 for controls and *n* = 20 for M‐AKTDKO mice). (C) Muscle weight of TA, EDL, soleus (SOL), and gastrocnemius (GAST) muscles from 8‐ to 12‐week‐old mice (*n* = 12 for controls and *n* = 20 for knockouts). (D) Wheat germ agglutinin (WGA) staining of EDL and soleus muscle cross sections (scale bar: 100 μm) and muscle fibre size distribution (*n* = 2). (E) Transcript level of atrogenes in gastrocnemius muscle of M‐AKTDKO mice compared with controls in *ad libitum* (*n* = 10–11) or 16 h fasted state (*n* = 3). (F) Proteolysis measured by tyrosine release in *ex vivo* muscle from control and M‐AKTDKO male mice (*n* = 4 for controls and *n* = 8 for M‐AKTDKO). (G) Proteasome activity in gastrocnemius lysates from control and M‐AKTDKO mice measured by breakdown of fluorescently labelled peptidyl glutamyl‐like (LLE) substrates (*n* = 4). (H) Puromycin labelling in M‐AKTDKO gastrocnemius muscles compared with the control muscles refed for 1 h following 16 h fasting (*n* = 4). (I) Protein fractional synthesis rate in M‐AKTDKO gastrocnemius muscles compared with the control muscles using deuterium incorporation rate over 36 h (*n* = 3–4). Representative experiment that was repeated in a separate cohort of mice (*P < 0.05, **P < 0.01, ^***^ *P* < 0.001, and ^****^ *P* < 0.0001 M‐AKTDKO vs. M‐Control; ^$$^ *P* < 0.01 and ^$$$$^ *P* < 0.0001 M‐Control (fed) vs. M‐Control (fasted); ^##^ *P* < 0.01, ^###^ *P* < 0.001, and ^####^ *P* < 0.0001 M‐AKTDKO (fed) vs. M‐AKTDKO (fasted); data are presented as mean ± SEM).

To gain a more in depth understanding of the role of AKT in muscle growth and function, we generated several new cohorts of mice with muscle‐specific ablation of AKT by crossing *AKT1*_*loxp/loxp*_ *;AKT2*_*loxp/loxp*_ mice with mice harbouring the HSA–CRE transgene to delete AKT expression in skeletal muscle (M‐AKTDKO).^12^ Western blot analysis of gastrocnemius muscle showed deletion of AKT2 (the predominant isoform of AKT present in skeletal muscles) using an AKT2‐specific antibody and a reduction in total AKT expression determined using a pan‐AKT antibody that recognized all AKT isoforms (*Figure* 1A). We previously verified that AKT expression is lost in all skeletal muscle depots while sparing cardiac and other metabolic tissues using this CRE transgene approach.^12^ Furthermore, while IGF‐1 phosphorylated well‐known targets of AKT such as pThr240 on PRAS40 and pSer240/244 on S6 and pSer256 on FOXO1 in M‐Control muscles, IGF‐1 failed to activate mTORC1 or inhibit FOXO1 in M‐AKTDKO mice as determined by the respective phosphorylation status of each protein (*Figure* 1A). These data are consistent with our previous work that showed a complete failure of insulin to phosphorylate S6, 4EBP1, PRAS40, FOXO1, and other known direct targets of AKT such as AS160, thereby confirming a functional loss of AKT activity in M‐AKTDKO muscle.^12^ Consistent with our previous findings, M‐AKTDKO mice showed a significant decrease in body weight (*Figure* 1B) paralleled by an ~40% decrease in muscle size isolated from different muscle depots such as tibialis anterior, gastrocnemius, EDL, and soleus muscles (*Figure* 1C). This was associated with a significant reduction in muscle weight‐to‐body weight ratio with the exception of soleus muscle (*Figure* S1A). This reduction in M‐AKTDKO muscle mass was associated with a significant reduction in muscle fibre size as documented by the wheat germ agglutinin staining of the CSA in EDL and soleus muscles of M‐Control and M‐AKTDKO mice (*Figure* 1D).

Changes in fibre size reflect an imbalance in proteostasis. Earlier studies suggest that AKT is not only capable of activating the protein synthesis pathway^4^ but is also important for suppressing protein degradation.^18^ Therefore, to gain insight into the mechanism of the reduced muscle fibre size in M‐AKTDKO muscles, we quantified rates of protein synthesis and degradation in muscle from M‐AKTDKO and M‐Control mice. Atrogenes such as *MURF‐1* and *Atrogin‐1* are highly up‐regulated in multiple models of muscle wasting and contribute to protein degradation.^18^ Therefore, we examined the expression levels of these genes in M‐AKTDKO and control muscles in the fed or 16 h fasted condition. As expected, fasting significantly increased most of the atrogenes expression compared with the fed condition in both genotypes; however, there was no difference between M‐Control and M‐AKTDKO muscles in many of the atrogenes including *Atrogin‐1* and *MURF‐1*. On the other hand, *p27* and *Gadd45a* expression trended to be elevated in M‐AKTDKO muscles compared to control muscles in the fed state (*Figure* 1E). Consistent with minimal changes observed with atrogene gene expression, rates of tyrosine release *ex vivo* and proteasome activity *in vivo* were unaltered or modestly reduced in M‐AKTDKO muscle compared with M‐Control muscle (*Figure* 1F and 1G). This was paralleled by no significant change in transcripts levels of proteasome subunits (*Figure* S1B) in M‐AKTDKO muscles compared with M‐Control muscles. Similarly, no appreciable increase in plasma or muscle amino acid levels was observed that would be predicted if the atrophy response was activated as seen in mice lacking IR/IGF1‐R specifically in skeletal muscle^19^ (*Figure* S1C and S1D). Moreover, no difference in the gene or protein expression of key autophagy markers such as *p62* and *LC3II/I* was observed in M‐AKTDKO muscles compared with controls in the *ad libitum* fed state (*Figure* S1E and S1F). While the ratio of protein expression of LC3II:I was increased in 16 h fasted control muscles compared with *ad libitum* fed control muscles, no appreciable increase in M‐AKTDKO muscles was observed compared with the control under fasted conditions (*Figure* S1E). Collectively, these data indicate that congenital deletion of skeletal muscle AKT reduces muscle mass and fibre size independent of multiple protein degradation pathways.

Next, we turned our attention to assessing the rates of muscle protein synthesis as a potential explanation for decreased muscle mass in M‐AKTDKO mice. While no change in the rates of fasting protein synthesis were observed between M‐Control and M‐AKTDKO muscles measured by the puromycin incorporation into total muscle protein in the 16 h fasted state (*Figure* S1G), animals refed for 1 h (to simulate protein synthesis) exhibit an ~25–30% decrease in protein synthesis in M‐ATKDKO mice compared with controls (*Figures* 1H and S1H). We confirmed these results using an orthogonal approach by measuring D_2_O incorporation to determine the fractional synthesis rate of muscle protein synthesis. Consistent with the puromycin labelling, the fractional synthesis rate following 36 h labelling with D_2_O water was similarly reduced in M‐AKTDKO muscles compared with control mice in both *ad libitum* or overnight fasted followed by 1 h refed mice (*Figures* S1I and 1L). Collectively, these data further confirm that AKT signalling in muscle is an important inducer of protein synthesis and muscle growth *in vivo*.

### Loss of AKT in muscle reduces exercise performance and contractile properties of EDL and soleus muscles

**Figure 2:**
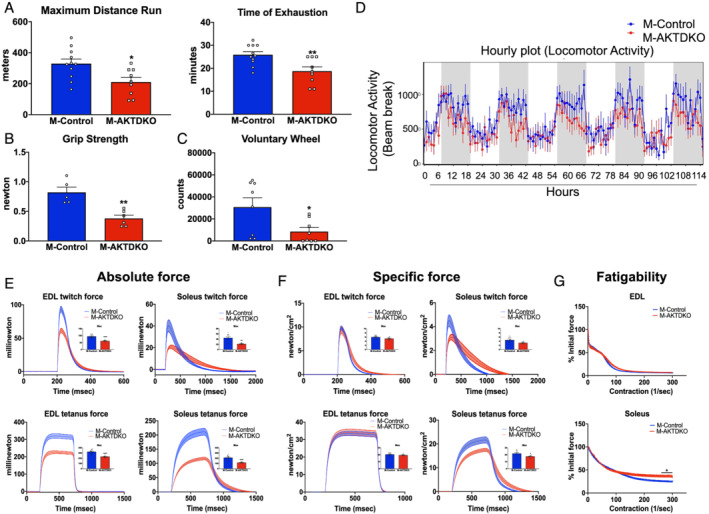
Loss of AKT in muscle reduces exercise performance and defects in contractile properties of EDL and soleus muscle. (A) Maximum distance run and time to exhaustion during an acute treadmill test of M‐AKTDKO and M‐Control mice (*n* = 9–11). (B) Forelimb grip strength in control and M‐AKTDKO mice (*n* = 5–6). (C) Cumulative number of revolutions during a voluntary wheel cage running experiment (*n* = 8). (D) Average daily activity measured over 5 days during the 12 h light and dark cycle (*n* = 6 for control and *n* = 5 for M‐AKTDKO). (E) Twitch and tetanic force and (F) specific force generated by isolated EDL and soleus muscles from M‐AKTDKO and M‐Control mice. Dashed lines indicated error bars. Inset: maximum force generated in M‐Control and M‐AKTDKO muscles (*n* = 11–12 for control and *n* = 6 for M‐AKTDKO). (G) Muscle fatigue in e*x vivo* contraction study with extensor digitorum longus (EDL) muscles or soleus muscles (*n* = 5–6) (*P < 0.05, **P < 0.01, and ^****^ *P* < 0.0001 vs. control; data are presented as mean ± SEM).

To address the functional consequences of AKT ablation in muscles, different exercise performance tests were performed in both genotypes. In a forced‐running acute treadmill test to measure exercise endurance, M‐AKTDKO mice performed poorly with an ~35% reduction in maximum distance run and ~30% decrease in running time compared with M‐Controls (*Figure* 2A). Muscle grip strength was reduced ~50% in M‐AKTDKO mice (*Figure* 2B). These effects were paralleled by the loss of voluntary wheel cage running activity quantified by a significant reduction in cumulative wheel counts over a week in M‐AKTDKO mice compared with M‐Controls (*Figure* 2C). No significant change in basal cage activity between M‐AKTDKO and M‐Control mice was observed as monitored through an automated Comprehensive Laboratory Animal Monitoring System. These results indicate that the poor exercise performance of M‐AKTDKO mice compared with M‐Control mice is likely independent of changes in locomotor activity (*Figure* 2D).

To determine if the poor exercise performance of M‐AKTDKO mice was solely due to a reduction in muscle mass or changes in the intrinsic property of the muscle itself, the contraction properties of muscles were studied in isolated EDL and soleus muscles *ex vivo*. The absolute maximal twitch and tetanic force in both EDL and soleus muscles were significantly lower in M‐AKTDKO mice compared with control muscles (*Figure* 2E). This decrease in absolute force capacity in EDL and soleus was due to a reduction in muscle mass because no difference in the specific maximal force was noted once normalized to the muscle CSA (*Figure* 2F). However, only a partial rescue in specific force generation in soleus muscle was observed, indicating a defect in the intrinsic properties of slow, oxidative fibres in muscle from M‐AKTDKO mice (*Figure* 2F). Finally, while EDL muscles isolated from M‐AKTDKO mice displayed similar rate of force dropping compared with control when subjected to repeated field electric stimulations, soleus muscles were resistant to fatigue (*Figure* 2G), suggesting a trade‐off between muscle strength and fatigue resistance in M‐AKTDKO soleus muscles. In summary, genetic deletion of skeletal muscle AKT decreases muscle mass coupled with defects in both *ex* vivo and *in vivo* muscle performance.

### Inhibition of FOXO1 and activation of mTORC1 alone are not sufficient to induce muscle growth in the absence of AKT

Studies over the last two decades using pharmacological or genetic inhibition of mTORC1 support a role for mTORC1 downstream of AKT in the regulation of skeletal muscle hypertrophy.^4^, ^10^, ^20^ To determine if activation of mTORC1 was sufficient to drive muscle growth in the absence of AKT, we generated mice lacking both AKT isoforms and TSC1 to drive mTORC1 signalling downstream of AKT (M‐AKTTSCTKO). Deletion of AKT2 and TSC1 was confirmed using western blot analysis in M‐AKTTSCTKO gastrocnemius muscles using specific antibodies (*Figure* S2A). Despite robust mTORC1 activation as documented by the enhanced pSer240/244 S6 and pThr37/46 4EBP1 (*Figure* S2A) following TSC1 deletion, M‐AKTTSCTKO muscles were significantly smaller as compared with their littermate controls with the exception of soleus muscles (*Figure* S2C).

In addition to mTORC1, the family of FOXO transcription factors regulate muscle mass.^9^, ^21^ Therefore, we next examined the role of FOXO1 acting downstream of AKT. Mice with combined deletion of both AKT isoforms and FOXO1 (M‐AKTFOXO1TKO) were generated to inhibit a major FOXO isoform in AKT‐deficient muscle. Western blot analysis in muscle lysates confirmed deletion of AKT2 and FOXO1 without causing up‐regulation of FOXO3a or activation of mTORC1 as determined by pSer240/244 S6 and pThr37/46 4EBP1 in M‐AKTFOXO1TKO muscles (*Figure* S2B). Similar to the selective activation of mTORC1, inhibition of FOXO1 alone was not sufficient to correct the loss in muscle mass of M‐AKTDKO mice as these mice had significantly smaller muscles in multiple muscle depots (*Figure* S2C). Collectively, these results provide *in vivo* evidence that neither activation of mTORC1 nor inhibition of FOXO1 alone was sufficient to rescue the muscle growth associated with the loss of AKT signalling. These data suggest that there is either redundancy between the two pathways or neither pathway alone is sufficient to drive muscle growth downstream of AKT.

### Coordinated inhibition of FOXO1 and activation of mTORC1 pathways rescue muscle mass and restore protein synthesis and body performance

**Figure 3:**
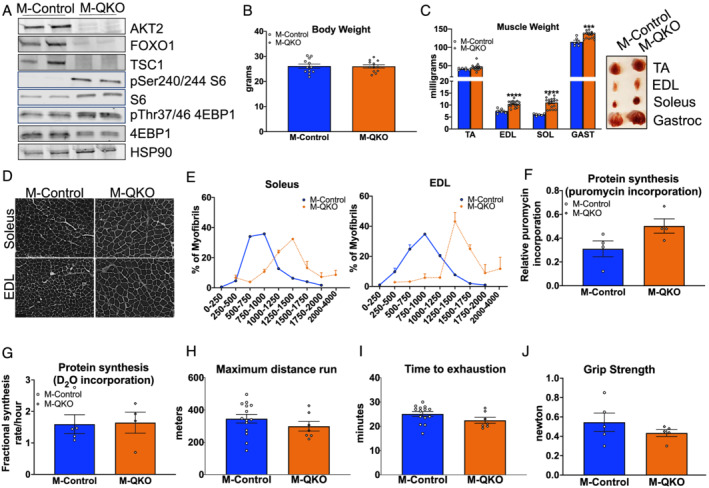
Simultaneous inhibition of FOXO1 and activation of mTORC1 are both required and sufficient to increase muscle mass and restore protein synthesis and performance in the absence of AKT. (A) Western blot of AKT2, FOXO1, TSC1, phosphor‐S6, S6, phospho‐4EBP1, total 4EBP1, and HSP90 in gastrocnemius muscle of control and M‐QKO mice. (B) Body weight (*n* = 12). (C) Muscle weight and representative figure of M‐QKO and M‐Control muscles of TA, EDL, soleus (SOL), and gastrocnemius (GAST) muscles from 8‐ to 12‐week‐old mice (*n* = 6 for controls and *n* = 18 for M‐QKO mice). (D) Wheat germ agglutinin (WGA) staining of EDL and soleus muscle cross sections (scale bar: 100 μm). (E) Myofibre size distribution (*n* = 3). (F) Puromycin labelling in M‐QKO gastrocnemius muscles compared with the control muscles (*n* = 4). (G) Protein fractional synthesis rate as measured by deuterium incorporation in the gastrocnemius muscles over 36 h (*n* = 4–5). (H) Maximum distance run and (I) time to exhaustion during an acute treadmill test of M‐QKO and M‐Control mice (*n* = 14 for controls and *n* = 7 for knockouts). (J) Forelimb grip strength in control and M‐QKO mice (*n* = 5) (^***^ *P* < 0.001 and ^****^ *P* < 0.0001 vs. M‐Control; data are presented as mean ± SEM).

Earlier *in vitro* studies suggest a role for mTORC1 in addition to FOXO transcription factors in the regulation of IGF‐1‐dependent transcriptional changes.^22^ Because activation of either pathway alone was insufficient to rescue muscle mass defect in M‐AKTDKO muscles, we hypothesized that simultaneous regulation of both FOXO1 and mTORC1 was required to mediate the AKT‐dependent effects on muscle size. Therefore, mice with combined deletion of both AKT isoforms, FOXO1, and TSC1 (M‐QKO) to simultaneously delete the FOXO1 pathway while activating mTORC1 in the absence of AKT were generated. Western blot analysis in gastrocnemius muscle confirmed the deletion of AKT2, FOXO1, and TSC1 and increased pSer240/244 S6 and pThr37/46 4EBP1 consistent with enhanced mTORC1 activity in gastrocnemius muscles from M‐QKO mice (*Figure* 3A). Phenotypic characterization revealed a complete restoration of body weight and muscle mass in M‐QKO mice as compared with their littermate controls (*Figure* 3B and 3C). Staining of muscle cross sections from M‐QKO EDL and soleus muscles with wheat germ agglutinin revealed complete rescue in the individual muscle fibre size (*Figure* 3D and 3E). This restoration of muscle mass correlated with the equivalent rates of protein synthesis compared with M‐Control muscles as measured by both puromycin incorporation and fractional synthesis rate in the refed state (*Figures* 3F, 3G, and S3A). Notably, this rescue in muscle mass seemed independent of the atrogenes such as MUSA1 and Gadd45a, whose transcript level was markedly increased in M‐QKO muscles similar to M‐AKTDKO muscles (*Figure* S3B). Finally, these improvements in muscle mass had functional consequences as, unlike M‐AKTDKO mice, M‐QKO mice exhibited normal exercise performance and grip strength similar to M‐Control mice (*Figure* 3H–3J). Collectively, these data support the hypothesis that combined inhibition of FOXO1 and activation of mTORC1 are both required and sufficient to regulate skeletal muscle fibre size and functional properties downstream of AKT.

### Loss of AKT induces changes in muscle morphology and a shift towards non‐oxidative fibre type via mTORC1 and FOXO1

**Figure 4:**
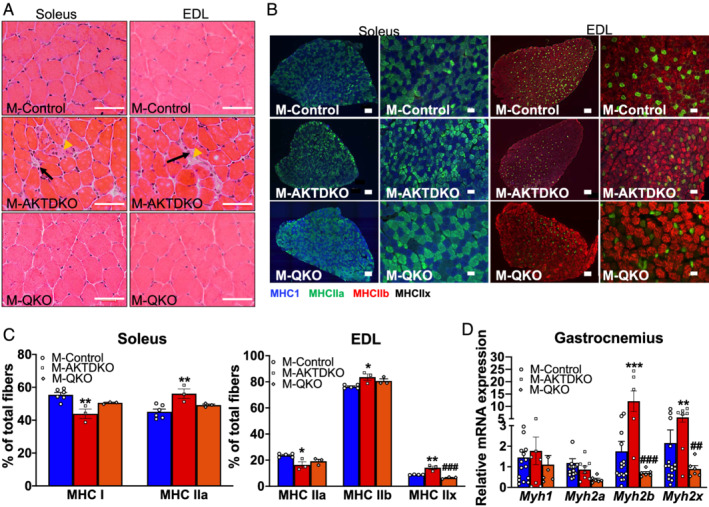
mTORC1 and FOXO1 act downstream of AKT to control muscle fibre‐type identity. (A) Hematoxylin and eosin (H&E) staining of muscle cross sections from the EDL and soleus muscles of 8‐ to 12‐week‐old mice (scale bar: 50 μm) from control, M‐AKTDKO, and M‐QKO mice. Both M‐AKTDKO EDL and soleus muscles contain unfiltered cells (black arrows) and fibrosis‐like structure (yellow arrowhead). (B) Immunostaining and (C) fibre‐type density (*n* = 3–4) for slow myosin heavy chain (MHC I; blue) and fast oxidative myosin heavy chain (MHC IIa; green) in soleus and for fast oxidative myosin heavy chain (MHC IIa; green), fast glycolytic myosin heavy chain (MHC IIb; red), and (MHC IIx; black) in EDL muscles (scale bar: 50 and 100 μm). (D) Gene expression of myosin heavy chain isoforms in gastrocnemius muscles in *ad libitum* state (*n* = 15 for controls, *n* = 8 for M‐AKTDKO muscles, and *n* = 6 for M‐QKO muscles) (*P < 0.05, **P < 0.001, and ^***^ *P* < 0.001 vs. control; ^##^ *P* < 0.01 and ^###^ *P* < 0.001 vs. M‐AKTDKO; data are presented as mean ± SEM).

The functional properties of muscles are determined in part by their fibre‐type composition. Therefore, we next sought to explore the direct contribution of AKT signalling *per se* on muscle physiology. Histological analysis and fibre composition of both soleus and EDL muscles were performed in M‐AKTDKO and M‐QKO muscle and compared with control muscles. Hematoxylin and eosin staining (H&E) revealed that loss of AKT induced signs of muscle myopathy, characterized by the alteration in size and shape of the fibres, muscle fibrosis (yellow arrowhead), and infiltration of macrophages (black arrows) (*Figure* 4A). Immunohistochemical staining using various MHC antibodies in EDL (glycolytic) and soleus (oxidative) muscle sections identified an ~35% increase in percentage of type IIa myofibres with a corresponding reduction in MHC I oxidative fibre density in the soleus of M‐AKTDKO mice (*Figure* 4B and 4C). Likewise, there was a significant reduction and increase in type IIa and type IIb/IIx fibres, respectively, in EDL of M‐AKTDKO mice (*Figure* 4B and 4C). To confirm our immunohistochemical analysis, gene expression of different isoforms of MHC in quadriceps, a mixed muscle, showed a significant increase in *MyHC IIb* and *MyHC IIx* mRNA (*Figure* 4D), confirming the fibre‐type switch towards a non‐oxidative type following loss of AKT. Notably, no difference in the basal activity of M‐AKTDKO and M‐Control mice (*Figure* 2D) suggests that the alteration in fibre composition in M‐AKTDKO muscles is independent of the changes in the locomotor activity of these mice. Thus, loss of AKT signalling induces a shift in fibre type towards a non‐oxidative muscle.

Next, we examined the downstream pathways via which AKT controls muscle fibre composition. Muscles isolated from different depots of M‐QKO hindlimbs appeared larger with a distinct ‘redder’ pigment (*Figure* 3C). This is in contrast to M‐AKTDKO muscles, which were smaller and appeared paler than the control muscles.^12^ Because changes in fibre type reflect a reprogramming of gene transcription leading to a remodelling of fibre contractile properties (slow–fast transitions) or metabolic profile (glycolytic–oxidative transitions), we determined the effect on muscle morphology and fibre composition in M‐QKO muscles. H&E staining performed in cross sections from EDL and soleus muscles of M‐QKO mice demonstrated that morphological structures were indistinguishable from that of the control muscles (*Figure* 4A). In addition, immunohistological analysis of both EDL and soleus muscles revealed no difference in the % type I or type IIa fibres in M‐QKO soleus or % type IIa and IIb fibres of M‐QKO EDL muscles compared with controls with a significant reduction in type IIx of M‐QKO EDL muscles compared with M‐AKTDKO EDL muscles (*Figure* 4B and 4C). This correlated with a significant reduction in the gene expression levels of type *IIb* and type *IIX* myosin isoforms in M‐QKO gastrocnemius compared with the M‐AKTDKO muscles (*Figure* 4D). Notably, immunohistochemical analysis of M‐AKTFOXO1TKO and M‐AKTTSCTKO EDL and soleus muscles displayed mild myopathy and fibre composition similar to M‐AKTDKO muscles, indicating that these individual pathways alone are not sufficient to drive the muscle structural properties in the absence of AKT (*Figure* S4A–S4C). Collectively, loss of muscle functional properties in association with the structural changes that occurred in response to genetic AKT deletion were completely reversed following the combined inhibition of FOXO1 and activation of mTORC1 activity.

### Deletion of AKT causes abnormalities in mitochondrial biogenesis, function, and morphology that are restored in M‐QKO mice

**Figure 5:**
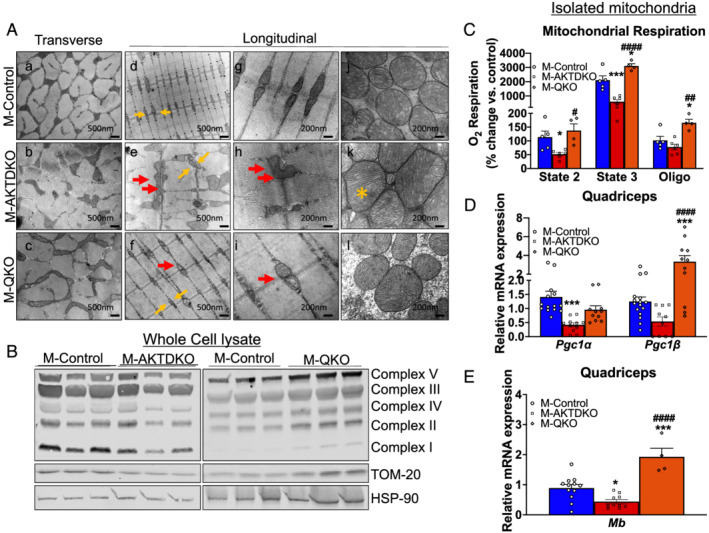
Abnormalities in mitochondrial biogenesis, function, and morphology in M‐AKTDKO mice are restored in M‐QKO mice. (A) Electron micrographs of transverse sections of gastrocnemius muscle from M‐AKTDKO and M‐QKO mice. Most intermyofibrillar mitochondria (yellow arrows) in M‐AKTDKO muscle are lost from their specific position next to Z disks (red arrows, panels e and h) and loss in cristae structure (yellow asterisk, panel k) that are restored in M‐QKO muscles. (B) Western blot for mitochondrial complex proteins, TOM‐20, and HSP90 in whole‐cell lysate of M‐AKTDKO and M‐QKO gastrocnemius muscles compared with M‐Controls. (C) Mitochondrial respiration capacity in the presence of NAD‐dependent (pyruvate/malate) substrates in freshly isolated mitochondria using high‐resolution respirometry from gastrocnemius muscle (*n* = 4–6). (D) Gene expression analysis in quadriceps muscles of M‐AKTDKO, M‐QKO, and M‐Control mice (*n* = 12 for controls and *n* = 11 for M‐AKTDKO and M‐QKOs). (E) Gene expression analysis of myoglobin in M‐AKTDKO and M‐QKO muscles compared with controls (*n* = 12 for controls and *n* = 4–10 for knockouts) (*P < 0.05 and ^***^ *P* < 0.0001 vs. control; ^#^ *P* < 0.05, ^##^ *P* < 0.01, and ^####^ *P* < 0.0001 vs. M‐AKTDKO; data are presented as mean ± SEM).

Mitochondria are unique organelles that provide energy for cell survival and are the sites of oxidative phosphorylation. When these organelles become dysfunctional, they produce less energy leading to muscle weakness and loss of endurance. To gain mechanistic insight into the reduced endurance capacity and the loss of oxidative phenotype in M‐AKTDKO muscles, we examined the effect on mitochondrial morphology and function. Ultrastructure analysis of the cross section of gastrocnemius muscles from M‐Control and M‐AKTDKO mice demonstrated a substantial loss of intermyofibrillar mitochondria in M‐AKTDKO muscle (*Figure* 5A, yellow arrow in panels d–f). This loss in mitochondrial content was accompanied by the swollen, disorganized mitochondria that are normally localized perpendicular to the Z disks (*Figure* 5A, red arrow in panels e, f, h, and i). Moreover, M‐AKTDKO mitochondria displayed morphological modifications characterized by the loss of parallel internal cristae (*Figure* 5A, yellow asterisk in panel k). We next determined the effect on mitochondrial functionality in M‐AKTDKO muscles compared with controls. In agreement with the ultrastructure analysis, western blotting performed in whole‐cell lysates revealed a significant reduction in mitochondrial complex proteins and TOM‐20 (a mitochondrial‐specific protein) expression, suggesting a reduction in total mitochondrial content (*Figure* 5B). Functionally, a significant reduction in State 1 and State 2 oxygen consumption rate was observed in mitochondria isolated from M‐AKTDKO skeletal muscle (*Figure* 5C). Conversely, these changes in mitochondrial content and morphology were completely rescued in M‐QKO muscles signifying the importance of both FOXO1 and mTORC1 pathways downstream of AKT in controlling mitochondrial morphology and content (*Figure* 5A and 5B). Further, mitochondrial oxygen consumption rate was significantly increased in M‐QKO mitochondria compared with the mitochondria isolated from M‐Control or M‐AKTDKO muscles (*Figure* 5C).

Mechanistically, these defects in mitochondrial biogenesis and function coincided with the down‐regulation of peroxisome proliferator‐activated receptor γ coactivator 1 (*Pgc1*α/β) expression (*Figure* 5D),^12^ a transcription factor that controls mitochondrial biogenesis and is a major regulator of oxidative metabolism in muscle and other tissues. Previous studies demonstrate that transgenic expression of *Pgc1*α in fast‐twitch muscle at or near physiological levels induced genetic programs characteristic of slow‐twitch muscle fibres.^23^ Moreover, this reduction in *Pgc1*α is associated with the significantly reduced transcript levels of myoglobin in M‐AKTDKO mice, a target *Pgc1*α gene in muscle (*Figure* 5E). Interestingly, M‐QKO normalized the mitochondrial biogenesis and muscle oxidative capacity in M‐AKTDKO muscles consistent with the rescue in *Pgc1* and myoglobin expression (*Figure* 5D and 5E). Collectively, these data indicate that mTORC1 and FOXO1 act synergistically to control muscle physiology and oxidative phenotype downstream of AKT in part by regulating *Pgc1*α/β expression. Together, these data provide genetic evidence for the critical role of AKT in maintaining skeletal muscle oxidative capacity and metabolism via coordination of both FOXO1 and mTORC1 pathways.

### Short‐term inducible knockout of AKT in adult skeletal muscle leads to a reduction in muscle mass and loss of oxidative properties

**Figure 6:**
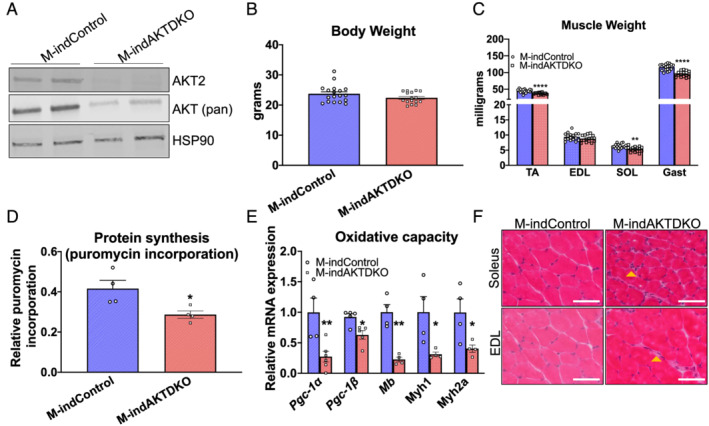
Short‐term inducible deletion of AKT in adult muscle leads to reduction in muscle mass and oxidative property. (A) Western blot for AKT2, AKT (pan), and HSP90 in quadriceps of M‐indControl and M‐indAKTDKO mice. (B) Body weight and (C) muscle weights of TA, EDL, soleus (SOL), and gastrocnemius (GAST) muscles of M‐indControl and M‐indAktDKO muscles (muscle inducible knockouts) harvested 5–7 weeks after the last day of tamoxifen injection (given for five consecutive days) (*n* = 18 for inducible controls and *n* = 16 for knockouts). (D) Puromycin labelling in M‐indAKTDKO gastrocnemius muscles compared with control muscles (*n* = 4). (E) Gene expression analysis in gastrocnemius muscles of M‐indAKTDKO and M‐Control (*n* = 4–6). (F) Hematoxylin and eosin (H&E) staining of muscle cross sections from the EDL and soleus muscles (scale bar: 50 μm) from control and M‐indAKTDKO mice (*P < 0.05, **P < 0.01, and ^****^ *P* < 0.0001 vs. M‐indControl; data are presented as mean ± SEM).

To examine the physiological relevance of AKT signalling in adult mice, we generated several mouse models of AKT deletion in adult skeletal muscle. Mice harbouring a CRE recombinase–oestrogen receptor fusion protein under the control of the HSA promoter (HSA–ESR–CRE) were crossed with the mice that contain *AKT1*_*loxp/loxp*_ *;AKT2*_*loxp/loxp*_ to induce an acute deletion of AKT1 and AKT2 (M‐indAKTDKO) in adult skeletal muscle. Western blot analysis indicated loss of AKT in M‐indAKTDKO muscles as determined by the AKT2 and AKT (pan) antibody (*Figure* 6A). As shown previously,^12^ M‐indAKTDKO mice displayed a significant ~15–20% reduction in muscle mass following 4–7 weeks post‐tamoxifen injections without significant loss in body weight (*Figure* 6B and 6C). No change in the atrogenes or autophagy mRNA expression was observed with the exception of *Gadd45a* whose levels were significantly up‐regulated in M‐indAKTDKO muscle (*Figure* S5A and S5B), similar to M‐AKTDKO muscle. Moreover, no difference in the proteasome activity or protein expression of the key autophagy markers was observed, suggesting that the loss in muscle mass in M‐indAKTDKO was independent of any of the proteolysis pathways (*Figure* S5C and S5D).

Consistent with M‐AKTDKO muscles, a significant reduction in the refed protein synthesis rate was observed M‐indAKTDKO muscles compared with controls as determined by the puromycin incorporation in 16 h fasted followed by 1 h refeeding (*Figures* 6D and S5E). In addition, the loss in muscle mass was associated with reduced oxidative gene expression such as *Pgc1*α/β and *Mb* (*Figure* 6E), similar to M‐AKTDKO muscles. Finally, H&E staining suggests mild myopathy characterized by mild infiltration of the immune cells (yellow arrow) (*Figure* 6F). Collectively, M‐indAKTDKO muscles recapitulate many of the hallmarks of the congenital (M‐AKTDKO) muscles, indicating that AKT signalling in adult muscle is an important determinant of muscle mass and the oxidative muscle phenotype.

### Inducible deletion of both FOXO1 and TSC1, but neither alone, is sufficient to reverse the loss muscle phenotype of M‐indAKTDKO mice

**Figure 7:**
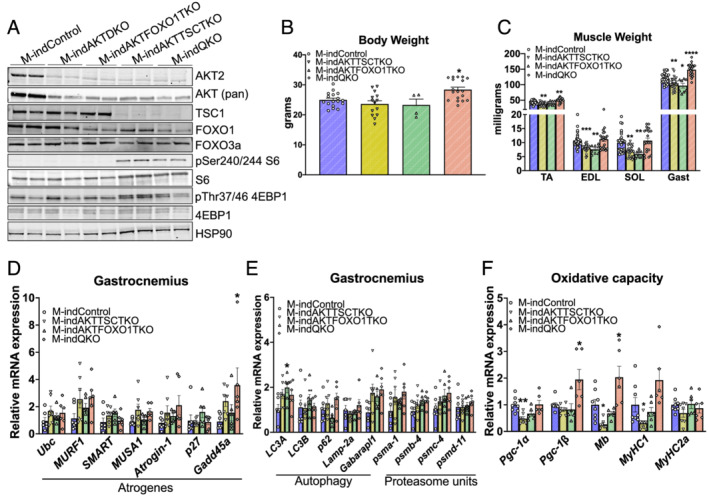
Deletion of both FOXO1 and TSC in adult skeletal muscles reverses muscle growth defect and oxidative capacity gene transcription. (A) Western blot for AKT2, AKT (pan), FOXO1, FOXO3a, TSC1, phospho‐S6, S6, phospho‐4EBP1, 4EBP1, and HSP90 in gastrocnemius of control and knockout mice. (B) Body weight and (C) muscle weights of TA, EDL, soleus (SOL), and gastrocnemius (GAST) muscles of M‐indControl and the knockout muscles (muscle inducible knockouts) harvested 4–7 weeks after the last day of tamoxifen injection (given for five consecutive days) (*n* = 30 for inducible controls and *n* = 8–26 for knockouts). (D, E) qPCR for mRNA of atrogenes, autophagy genes, and proteasome subunit genes in gastrocnemius muscle from control, M‐indAKTFOXO1TKO, M‐indAKTTSCTKO, and M‐indQKO mice in *ad libitum* condition (*n* = 6–7). (F) Relative mRNA levels of PGC1, myoglobin (Mb), MyHC1, and MyHC2a in gastrocnemius muscle 4 weeks after tamoxifen treatment (*n* = 6–8) (*P < 0.05, **P < 0.01, ^***^ *P* < 0.001, and ^****^ *P* < 0.0001 vs. control; data are presented as mean ± SEM).

To explore if both FOXO1 and mTORC1 pathways are necessary downstream of AKT to control adult skeletal muscle physiology and to rule out any developmental complications associated with chronic knockout from birth, we crossed mice harbouring HSA–ESR–CRE with *AKT1*_*loxp/loxp*_ *;AKT2*_*loxp/loxp*_ *;FOXO1*_*loxp/loxp*_, *AKT1*_*loxp/loxp*_ *;AKT2*_*loxp/loxp*_ *;TSC1*_*loxp/loxp*_, or *AKT1*_*loxp/loxp*_ *;AKT2*_*loxp/loxp*_ *;FOXO1*_*loxp/loxp*_ *;TSC1*_*loxp/loxp*_ mice to induce acute deletion of AKT–FOXO1 (M‐indAKTFOXO1TKO), AKT–TSC1 (M‐indAKTTSCTKO), or AKT–FOXO1–TSC1 (M‐indQKO). Western blot analysis confirmed the deletion of AKT in all knockout genotypes, FOXO1 in M‐indAKTFOXO1TKO/M‐indQKO, and TSC1 in M‐indAKTTSCTKO/M‐indQKO muscle (*Figure* 7A). Moreover, M‐indAKTTSCTKO/M‐indQKO muscle extracts showed the expected increase in the mTORC1 markers pSer240/244 S6 and pThr37/46 4EBP1. While no marked difference in body weights of M‐indAKTFOXO1TKO and M‐indAKTTSCTKO mice was observed, M‐indQKO mice were significantly heavier than the control mice (*Figure* 7B). In agreement with our congenital mice results, all the muscles are significantly smaller in M‐indAKTFOXO1TKO and M‐indAKTTSCTKO mice, which were completely normalized in M‐indQKO mice (*Figure* 7C). Despite significant loss in muscle mass, similar to M‐indAKTDKO, no change in the transcripts of atrogenes (including *atrogin‐1*), autophagy factors, or proteasome subunits was observed in the M‐indAKTFOXO1TKO or M‐indAKTTSCTKO muscles, with the exception of *LC3A*, which trended to be higher in M‐indQKO muscles, again suggesting that the rescue in muscle mass was independent of protein degradation genes (*Figure* 7D and 7E). Strikingly, the mRNA expression of *Pgc1α*, *Mb*, and *MyHC1* was completely rescued in M‐indQKO muscles but not following activation of mTORC1 or inhibition of FOXO1 alone (*Figure* 7F). These results are consistent with the phenotyping characterization of M‐AKTDKO mice and the rescue in M‐QKO, which together support the notion that AKT maintains skeletal muscle oxidative capacity and metabolism via coordinated regulation of both FOXO1 and mTORC1 pathways but neither pathway alone.

### Inducible activation of mTORC1 and inhibition of FOXO1 in adult skeletal muscles prevent immobilization‐induced muscle wasting

**Figure 8:**
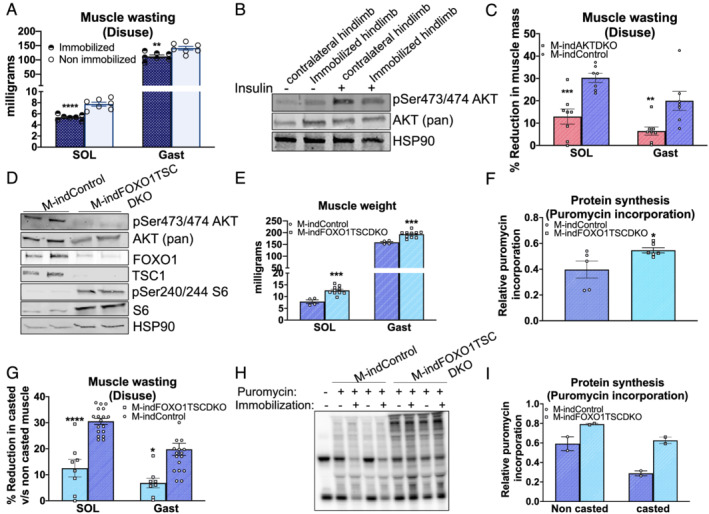
Inducible deletion of FOXO1 and TSC1 prevents immobilization‐induced muscle wasting in adult skeletal muscle. (A) Muscle mass of immobilized leg compared with non‐immobilized leg of control mice (*n* = 7). (B) Western blots for phospho‐AKT, total AKT (pan), and HSP90 protein in gastrocnemius muscles from immobilized vs. non‐immobilized leg injected with or without insulin (2 U/kg body weight) following overnight fasting. (C) Percent reduction in muscle mass of the leg casted for 7 days vs. the contralateral non‐casted leg in control and M‐AKTDKO mice (*n* = 6 for controls and *n* = 8 for knockout). (D) Western blot of phospho‐AKT, AKT (pan), FOXO1, TSC1, phospho‐S6, S6, and HSP90 in quadriceps of M‐indControl and M‐indFOXO1TSCDKO mice. (E) Soleus (SOL) and gastrocnemius muscles (GAST) muscles of M‐indControl and M‐indFOXO1TSCDKO muscles (muscle inducible knockouts) harvested 4 weeks after the last day of tamoxifen injection (given for five consecutive days) (*n* = 4 for inducible controls and *n* = 10 for M‐indFOXO1TSCDKOs). (F) Puromycin labelling in M‐FOXO1TSCDKO gastrocnemius muscles compared with the control muscles (*n* = 5–6). (G) Per cent reduction in muscle mass of the leg casted for 7 days vs. the contralateral non‐casted leg (*n* = 18 for controls and *n* = 8 for knockout). (H) Puromycin labelling and (I) quantification in gastrocnemius muscles of the leg casted for 7 days vs. the contralateral non‐casted leg in control and M‐FOXO1TSCDKO mice (*n* = 2) (*P < 0.05, **P < 0.01, ^***^ *P* < 0.001, and ^****^ *P* < 0.0001 vs. control; data are presented as mean ± SEM).

Defective insulin/IGF‐1 signalling via AKT is associated with several models of muscle wasting.^4^, ^5^, ^24^ Additionally, evidence from human and animal studies clearly show that a shift in slow‐to‐fast myosin isoforms combined with reduced protein synthesis are characteristic features of several models of muscle wasting including disuse/immobilization drawing stark similarities to the genetic loss of AKT.^25^ Therefore, we next sought to understand the underlying mechanisms of disuse‐induced muscle wasting and define the specific role of the AKT‐dependent signalling in this response. We employed the well‐characterized immobilization model of disuse‐induced muscle wasting to stimulate a robust and physiological reduction in muscle mass that is accompanied with defects in protein synthesis and a fibre‐type shift.^15^ We confirmed a significant, ~20–30%, reduction in soleus and gastrocnemius muscle mass (the muscles most affected in response to immobilization)^26^ following 7 days of casting (*Figure* 8A). Consistent with previous reports, this was associated with a decrease in the insulin‐stimulated phosphorylation status of Ser473/474 AKT in mouse gastrocnemius muscle (*Figure* 8B). We next examined if a reduction in AKT activity was critical to the muscle wasting following disuse. Interestingly, while immobilization of control muscles caused a 20–30% reduction in soleus and gastrocnemius muscle mass, this approach failed to induce a similar decrease in muscle mass in M‐indAKTDKO mice, indicating that this model is dependent, in part, on a reduction in AKT activity to drive muscle loss (*Figure* 8C).

To test the hypothesis that decreased AKT signalling to both mTORC1 and FOXO1 pathways contributes muscle loss in response to disuse, we generated mice with combined deletion of FOXO1 and TSC1 by crossing *FOXO1*_*loxp/loxp*_ *;TSC1*_*loxp/loxp*_ mice with mice harbouring HSA–ESR–CRE to induce an acute deletion of FOXO1 and activation of mTORC1 without genetically perturbing AKT (M‐indFOXO1TSCDKO). M‐indFOXO1TSCDKO mice displayed loss of FOXO1 and TSC1 and enhanced mTORC1 activity as documented by increased pSer20/244 S6 (*Figure* 8D). M‐indFOXO1TSCDKO muscles were significantly bigger than their floxed controls (*Figure* 8E), with no change in body weight (*Figure* S6A). This increase in muscle mass was associated with a marked increase in protein synthesis rate (*Figures* 8F and S6B) with minimal effects on the expression of atrogenes, autophagy factors, or proteasome subunit genes with the exception of *MURF1* and *p62* (*Figure* S6C). Despite the up‐regulation of these genes, the complete rescue in the muscle mass concurrent with the enhanced protein synthesis rate in M‐indFOXOTSCDKO muscles indicated that defects in protein synthesis are a major driving factor of muscle mass in the absence of AKT signalling. Finally, casting one of the hindlimbs of M‐indFOXO1TSCDKO mice for 7 days significantly attenuated the immobilization‐induced muscle loss in both soleus and gastrocnemius muscles (*Figure* 8G). This coincided with an increase in the rate of protein synthesis in M‐indFOXO1TSCDKO casted muscles compared with the control casted muscles (*Figure* 8H and 8I). In summary, these data identified the specific distal mechanisms that are both required and sufficient to mediate AKT's positive effects on muscle growth and function *in vivo*. Moreover, mimicking AKT signalling by coordinated regulation of mTORC1 and FOXO1 improved muscle mass in response to a physiological stressor such as disuse‐induced muscle wasting.

## Discussion

The role of the serine/threonine kinase AKT in regulating skeletal muscle growth is highly appreciated. Earlier *in vivo* studies involving transient transfection of a constitutively active AKT1^4^ or constitutively active inducible AKT1 transgene^16^, ^27^ in skeletal muscles demonstrate a striking effect of AKT1 on muscle size, highlighting the important role of AKT in regulating muscle mass. Whole‐body knockout of both AKT isoforms results in organismal growth retardation and early postnatal lethality.^28^ Our previous work identified that individual deletion of skeletal muscle AKT1 or AKT2 is insufficient to drive a muscle growth defect because of residual AKT signalling from either isoform.^12^ Here, we demonstrate that autonomous AKT signalling is required for muscle mass maintenance of both developing and adult muscles. Moreover, we elucidate a new role of AKT in the control of the muscle oxidative metabolism, muscle performance, and muscle function. Importantly, we map the key distal signalling pathways mediating these anabolic effects of AKT. Specifically, we show that AKT coordinately activates mTORC1 and inhibits FOXO1 to preserve muscle mass and function in response to genetic AKT deficiency and immobilization‐induced muscle wasting. These data highlight the complex molecular crosstalk underlying muscle fibre size and fibre‐type specification and establish a role for AKT signalling in immobilization‐induced muscle wasting via downstream mTORC1 and FOXO1 signalling.

In contrast to the current popular view of the dual regulation of protein synthesis and degradation, we find that AKT controls muscle mass in both developing and adult muscles primarily via muscle protein synthesis without major changes in muscle proteolysis and atrogene gene expression (*Figures* 1, S1, 6, and S5). Surprisingly, activation of mTORC1 alone, the canonical signalling node implicated in protein synthesis, was not sufficient to restore muscle mass following AKT deletion (*Figures* S2 and 7). This is consistent with the previous literature that suggests sustained activation of mTORC1 following genetic deletion of *TSC1* in skeletal muscle (TSCmKO mice) is not sufficient to increase muscle mass and in fact induces modest muscle loss in several muscle depots.^11^ Moreover, loss of Raptor in adult muscle does not lead to a reduction in muscle mass, suggesting that AKT‐dependent, mTORC1‐independent pathways are critical for the maintenance of muscle mass *in vivo*.^29^ Interestingly, a direct role of FOXO proteins on protein synthesis has been suggested in *Drosophila* muscle through activation of mRNA translation repressor eIF4E‐binding protein (4E‐BP)^30^, ^31^ and TSC‐2/sestrin‐mediated mTORC1 suppression.^32^, ^33^ Notably, our data indicate an important role for the AKT–FOXO1 axis in addition to mTORC1 in the control of muscle proteostasis. Specifically, our data show that ablation of FOXO1, a predominant skeletal muscle isoform of FOXO regulating muscle mass that is induced in some forms of muscle wasting,^21^, ^34^ along with the activation of mTORC1 are both required and sufficient to regulate muscle growth and protein synthesis downstream of AKT (*Figures* 3 and 7). Therefore, the insulin/IGF‐1 signalling network responsible for muscle homeostasis appears to be more complex than a simple AKT–mTORC1‐dependent anabolic vs. AKT–FOXO‐dependent catabolic mechanism.

Growing evidence implicates the activation of the proteasome degradation system in muscle loss that is, in part, mediated through the activation of FOXO transcription factors.^9^ Most recently, mice lacking IR and IGF‐1R (MIGIRKO) were shown to induce muscle loss in a FOXO‐dependent manner. Paradoxically, loss of IR/IGF‐1R in skeletal muscle up‐regulates AKT and mTORC1 activity.^8^ Thus, reducing IR/IGF‐1R clearly prevents insulin action in skeletal muscle; however, downstream signalling via AKT is still active, implicating an AKT‐independent mechanism of FOXO activation leading to muscle loss. Our data demonstrate that reduced fibre size following AKT depletion is independent of the protein degradation pathway as there was no evidence of increased proteolysis or enhanced amino acid breakdown in response to genetic ablation of AKT (*Figure* S1). Likewise, no significant effect on gene expression of the atrogenes was observed in M‐AKTDKO and M‐indAKTDKO mice except for a consistent change in *Gadd45a* (*Figures* 1 and S5). Previous reports have identified *Gadd45a* as an important factor in inducing skeletal muscle atrophy.^35^ However, the enhanced expression of *Gadd45a* is not likely to be driving muscle loss following AKT deficiency as the mRNA expression was not restored in both M‐QKO or M‐indQKO muscles despite complete rescue in muscle mass (*Figures* S3 and 7). These data support a *Gadd45a*‐independent mechanism for muscle loss following the inhibition of AKT signalling *in vivo*.

It is worth noting that muscles lacking both IR and IGF‐1R^8^ as well muscles from various chronic muscle atrophy model in both humans and mice display no change or even a decrease in several atrogenes such as *Atrogin‐1* and *MURF‐1*.^8^, ^36^, ^37^ These results suggest that a reduction in signals in addition to AKT is required to induce *Atrogin‐1* and *MURF‐1* gene expression. One possible explanation is that there is still some degree of basal mTORC1 activity in M‐AKTDKO muscle, which is capable of restraining the induction of the key atrogenes.^22^, ^38^ In addition, we cannot rule out the possibility that the dependence on protein synthesis is unique to AKT deletion from birth; however, we observed a similar phenotype on muscle mass in our adult inducible knockout system (*Figures* 6 and S5). Importantly, these data do not imply that AKT‐dependent control of muscle atrogene expression is not important under certain pathological conditions, which are well documented.^18^ Rather, these data emphasize the importance of muscle AKT signalling in the control of protein synthesis, oxidative metabolism, and muscle performance.

Functionally, lower muscle endurance, exercise intolerance, and reduced susceptibility to fatigue are characteristic features of atrophied muscle. Despite this association, it is important to note that muscle force development is not always associated with muscle mass. Thus, while AKT signalling plays an important role in skeletal muscle hypertrophy,^3^, ^4^ the functional and physiological aspects of this muscle growth remain ill defined. Moreover, recent models of targeted gene knockout of reduced insulin and insulin‐like growth factor signalling via their respective receptors failed to clarify this conundrum as these genetic models have not identified the mechanism of reduced oxidative and endurance capacity in association with muscle loss. Notably, loss of both IR and IGF1R in muscles induces a shift towards the oxidative type, where paradoxical increase in AKT and mTORC1 is observed because of an uncharacterized negative feedback loop.^8^ Notably, loss of mTORC1 signalling following *raptor* deletion also induces a shift towards an oxidative fibre type.^10^ Our study demonstrated that AKT‐deficient skeletal muscles display a substantial reduction in *Pgc1α/β* expression with the loss in mitochondrial function (*Figures* 5 and 6).^12^ This is in agreement with earlier work by Das *et al*. who demonstrated that IGF‐1 regulates mitochondrial function in skeletal muscle in an AKT‐dependent manner.^5^ Additionally, we show that inhibition of insulin signalling via AKT in skeletal muscle *per se* causes a shift towards non‐oxidative muscle (*Figures* 4 and 6), loss in mitochondrial ultrastructure, content, and function (*Figure* 5),^12^ with the concomitant loss in exercise performance (*Figure* 3). These are consistent with the molecular features of Pgc1α/β knockout muscles^23^, ^39^, ^40^, ^41^ suggesting the critical role of AKT in regulating Pgc1α/β transcripts to maintain muscle oxidative capacity and function. Mechanistically, our study identified that AKT signalling via FOXO1 and mTORC1 activity regulates muscle oxidative metabolism (*Figures* 4, S4, 5, and 7). Transgenic expression of *Pgc1α* in mTORC1‐deficient muscles rescues muscle oxidative capacity and mitochondrial function.^42^ Thus, the rescue in *Pgc1*α/β expression in M‐QKO and M‐indQKO muscles (*Figures* 5 and 7) provides further evidence that the mitochondrial deficits and loss in muscle oxidative capacity in M‐AKTDKO mice are likely due to a transcriptional reduction in *Pgc1α/β* expression that is regulated by both FOXO1 and mTORC1 pathways. While our study established the AKT‐mediated pathways that are required and sufficient to regulate muscle oxidative capacity, more work is needed to understand the underlying transcriptional mechanisms and hierarchy responsible for this effect.

Muscle wasting, also referred to as muscle atrophy, is assumed to show a combination of decreased muscle protein synthesis and increased protein breakdown rate.^43^ Yet the individual contribution of decreased muscle protein synthesis may differ between various types of atrophies. For example, in disuse‐induced muscle wasting, sarcopenia, cachexia, sepsis, and so forth, blunted muscle protein synthesis rates seem to be the predominant cause for a decline in muscle mass,^44^, ^45^, ^46^ indicating a potential benefit of interventions, which increase muscle protein synthesis in these muscles. Although in most cases changes in mTORC1 signalling and phosphorylation of its downstream targets correlate well with alterations in muscle protein synthesis, in some studies, modulation of upstream regulators of mTORC1 signalling exhibits inconsistent patterns of change. Moreover, recent work by You *et al*. highlights the role of pathways in addition to mTORC1 that are required to mitigate immobilization‐induced muscle wasting.^15^, ^47^ Thus, a more detailed understanding of the regulatory mechanisms controlling protein synthesis will aid in the development of effective and specific therapies that preserve muscle mass.

Reduced AKT activity is observed in several different models of muscle wasting.^5^, ^24^ Importantly, AKT signalling appears to be crucial in recovery of muscle mass following unloading or hindlimb suspension.^16^ However, despite these correlative studies, the molecular mechanisms underlying disuse‐induced muscle loss and which AKT‐dependent signalling pathways regulate this effect *in vivo* remain unresolved. Our study identifies that both mTORC1 and FOXO1 pathways are critical components of muscle wasting in response to disuse (*Figure* 8). Importantly, because various types of atrophy share a common set of transcriptional adaptations, it is tempting to speculate that the protective role of AKT in disuse‐induced muscle loss may extend to other models such as sarcopenia that also have reduced muscle AKT activity.^5^ Future studies will test the role of this AKT‐dependent signalling in other models of muscle atrophy including denervation and ageing. Of note, the present study does not mitigate the role of other AKT‐dependent–mTORC1‐independent pathways such as glycogen synthase kinase‐3, which has been shown to control muscle proteostasis.^48^ However, recent studies question the specific role of the AKT‐mediated phosphorylation of GSK3 in maintaining muscle mass under basal and disuse conditions.^49^ Rather, the goal of this study was to map the AKT‐dependent pathways that are both necessary and sufficient to control muscle mass and oxidative metabolism and define their specific contribution to disuse‐induced atrophy.

In summary, our work demonstrates that AKT is an obligate intermediate in the insulin/IGF‐1 signalling pathway that controls muscle mass through coordination of both FOXO1 and mTORC1 pathways. Additionally, by mimicking distal AKT signalling genetically, the muscle wasting associated with disuse can be dramatically prevented. Future studies will continue to explore the proximal and distal signalling mechanisms governing muscle hypertrophy, atrophy, and fibre‐type specification, which will be essential for our mechanistic understanding of muscle physiology in health and disease.

## Funding

This work was supported by the US National Institutes of Health Grants DK123252 (P.M.T.), DK15658 (S.R.K), and F32DK126312 (P.A.R.); P&F grant Penn Diabetes Research Center DK19525 (P.M.T.); the Samuel and Josephine Chiaffa Memorial Fund (P.M.T.); and Cox Research Institute and institutional start‐up funds from the University of Pennsylvania.

## Conflict of interest

None declared.

## Supporting information


**Figure S1.** Deletion of AKT in skeletal muscle does not affect amino acid levels or upregulate autophagy (a) Muscle weight is to body weight ratio in M‐AKTDKO muscles compared to control muscles (n = 12 for controls and *n* = 20 for knockouts) (b) qPCR of proteasome subunits in gastrocnemius muscle of M‐AKTDKO mice compared to controls in *ad libitum* state (n = 5–7) (c and d) Essential amino acid levels, BCAA and aromatic amino acid levels measured by metabolomic analysis in gastrocnemius muscles of M‐AKTDKO mice compared to M‐Control mice (n = 5) and in plasma using mass spectrometry (n = 6–7) (e) Western blots for autophagy intermediates in gastrocnemius from fed or 16 h fasted M‐AKTDKO vs M‐Control with densitometric analysis of p62 or ratio of LC3‐II/LC3‐I normalized to HSP90 (f) Relative mRNA expression of autophagy genes in gastrocnemius muscle of M‐AKTDKO mice compared to controls in *ad libitum* state (n = 5–12) (g) Representative blot and quantification for puromycin incorporation in gastrocnemius muscles following 16 h fasting in M‐indAKTDKO muscles (n = 4–5) (h) Representative blot for puromycin labelling in M‐AKTDKO gastrocnemius muscles compared to the control muscles refed for 1 h following 16 h fasting (n = 4) (i) Protein fractional synthesis rate in M‐AKTDKO gastrocnemius muscles compared to the control muscles using deuterium incorporation rate over 36 h. Muscles were harvested following fasting for the last 16 h (n = 4–5) (*P < 0.05, **P < 0.01, ****P < 0.0001 vs. control, data are presented as mean ± SEM).
**Figure S2.** Inhibition of FOXO1 and activation of mTORC1 alone are not sufficient to induce muscle growth in the absence of AKT (a) Western blot of AKT2, FOXO1, phospho‐S6, S6, phospho‐4EBP1 and HSP90 in of M‐AKTTSCTKO and (b) M‐AKTFOXO1TKO gastrocnemius muscle compared to their floxed littermates. (c) Muscle weights of TA, EDL, soleus (SOL) and gastrocnemius (GAST) muscles of M‐Control and knockout mice (n = 18 for controls, n = 16 for M‐AKTTSCTKO and *n* = 40 for M‐AKTFOXO1TKO mice) (**p < 0.01 and **** p < 0.0001 vs. M‐Control, data are presented as mean ± SEM).
**Figure S3.** qPCR analysis of atrogenes (a) Representative blot for puromycin labelling in M‐QKO gastrocnemius muscles compared to the control muscles refed for 1 h following 16 h fasting (n = 4) (b) Relative mRNA expression of the atrogenes in M‐QKO muscles compared to the control muscles (*n* = 7–8) (* *p* < 0.05, **p < 0.01 and ***p < 0.001 vs. M‐Control, data are presented as mean ± SEM).
**Figure S4.** Individual deletion of FOXO1 and TSC pathway is not sufficient to rescue the fiber type switch in M‐AKTDKO muscles (a) Hematoxylin and Eosin (H&E) staining of muscle cross‐sections from the EDL and soleus muscle (scale bar: 50 μM) from control, M‐AKTFOXTKO and M‐AKTTSCTKO mice (b) Immunostaining and (c) fiber density (n = 3) for slow myosin heavy chain (MHC I; blue) and fast oxidative myosin heavy chain (MHC IIa; green) in soleus and for fast oxidative myosin heavy chain (MHC IIa; green), fast glycolytic myosin heavy chain (MHC IIb; red) and (MHC IIx; black) in EDL muscles (scale bar: 100 μM) (*p < 0.05, **p < 0.01, ***p < 0.001 vs. M‐Control, data are presented as mean ± SEM).
**Figure S5.** Inducible loss of AKT in adult muscle and effects on gene expression for protein degradation pathways (a and b) Transcript level of atrogenes, autophagy genes and proteasome subunits in gastrocnemius muscle of M‐indAKTDKO mice compared to controls in *ad libitum* state (n = 4) (c) Proteasome activity in gastrocnemius lysates from control and M‐indAKTDKO mice measured by breakdown of fluorescently labeled peptidyl glutamyl‐like (LLE) substrates (n = 4) (d) Western blots for autophagy intermediates in gastrocnemius from M‐AKTDKO vs M‐Control with densitometric analysis of p62 or ratio of LC3‐II/LC3‐I normalized to HSP90 in *ad libitum* condition (e) Representative blot for puromycin labelling in M‐indAKTDKO gastrocnemius muscles compared to the control muscles refed for 1 h following 16 h fasting (n = 4) (**p < 0.01 vs. M‐Control, data are presented as mean ± SEM).
**Figure S6.** M‐indFOXO1TSCDKO exhibit no change in body weight or atrophy, autophagy, and proteasome subunit gene expression (a) Body weight of M‐indFOXO1TSCDKO mice compared to controls (n = 7–9) (b) Representative blot for puromycin labelling in M‐indFOXOTSCDKO gastrocnemius muscles compared to the control muscles refed for 1 h following 16 h fasting (n = 4) (c‐d) qPCR of atrogenes, autophagy genes and proteasome subunits genes in gastrocnemius muscles in *ad libitum* condition relative to controls in (c) Non‐immobilized and (d) immobilized M‐indFOXOTSCDKO vs control muscles (n = 4–5) (*p < 0.05, **p < 0.01 vs. M‐Control, data are presented as mean ± SEM)
