# Supplementary figures and images for: AKT controls protein synthesis and oxidative metabolism via combined mTORC1 and FOXO1 signalling to govern muscle physiology
Source: J Cachexia Sarcopenia Muscle. 2021 Nov 9;13(1):495–514. doi: 10.1002/jcsm.12846 (PMC8818654; doi:10.1002/jcsm.12846)

Figure S1

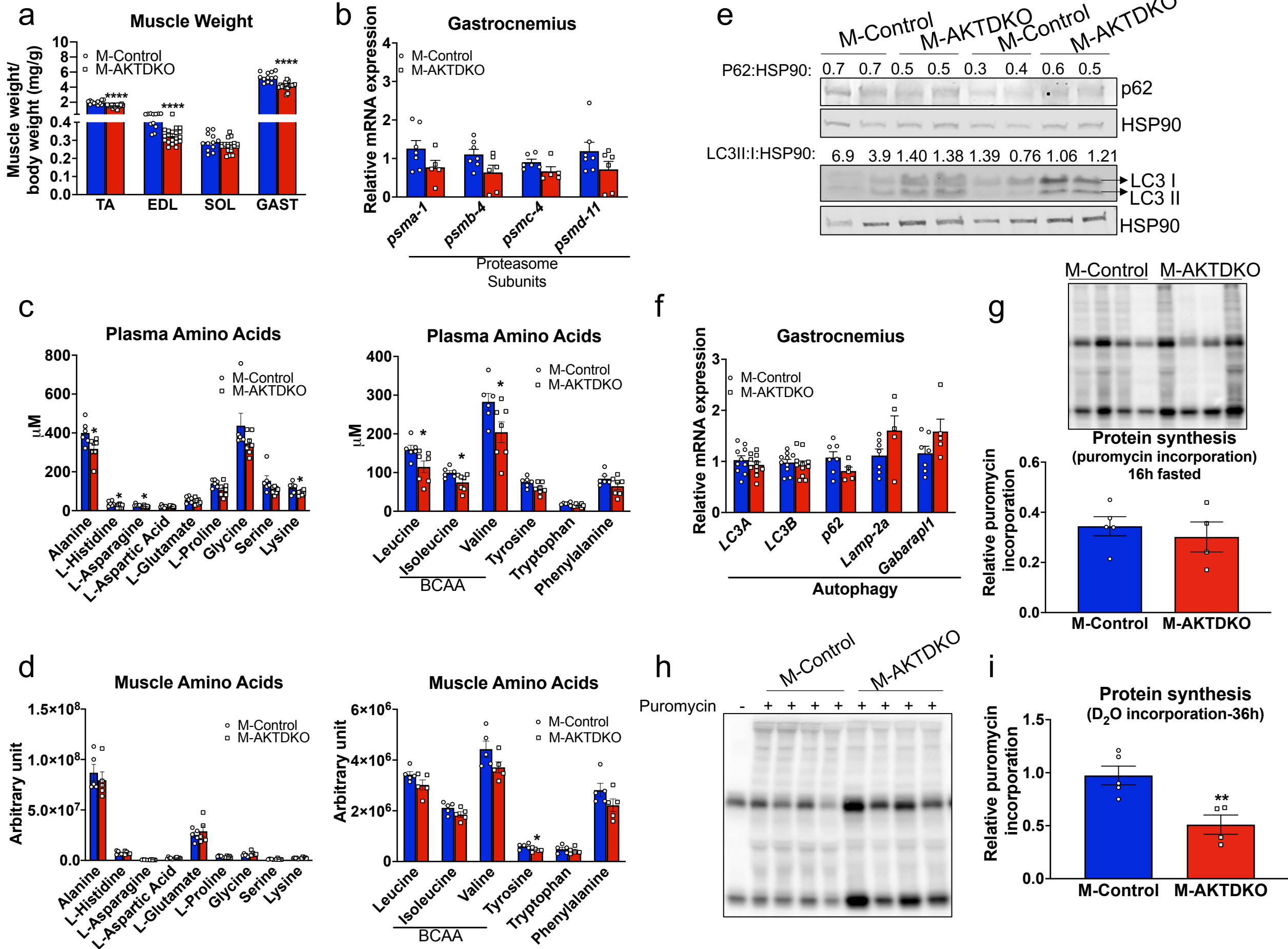

Figure S2

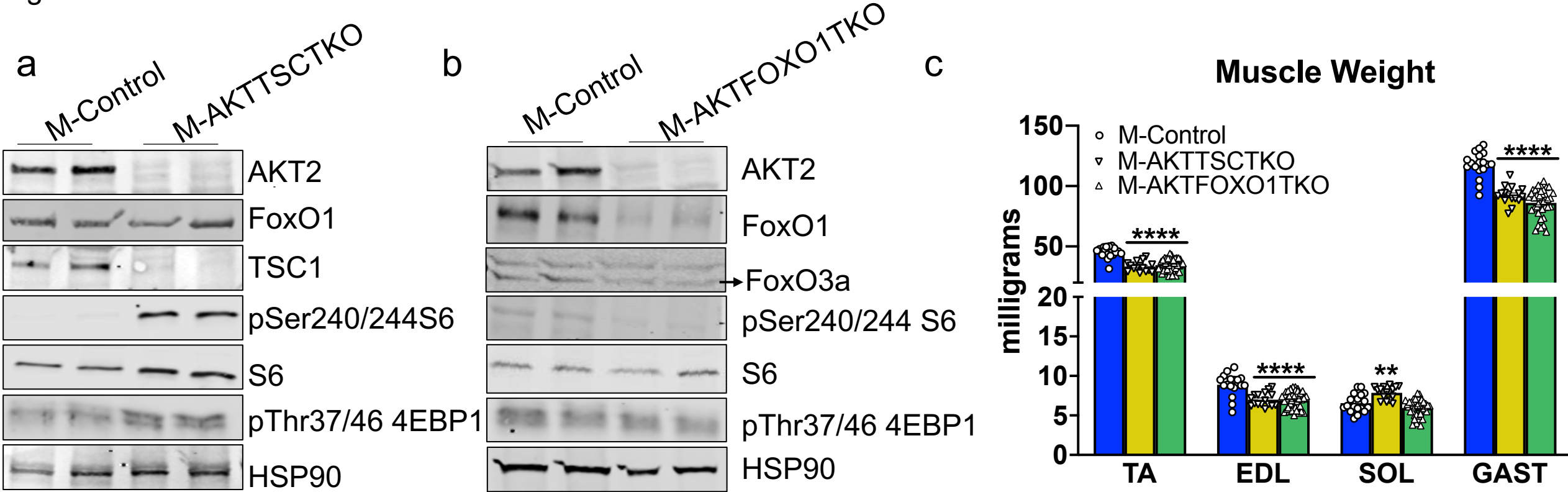

Figure S3

a

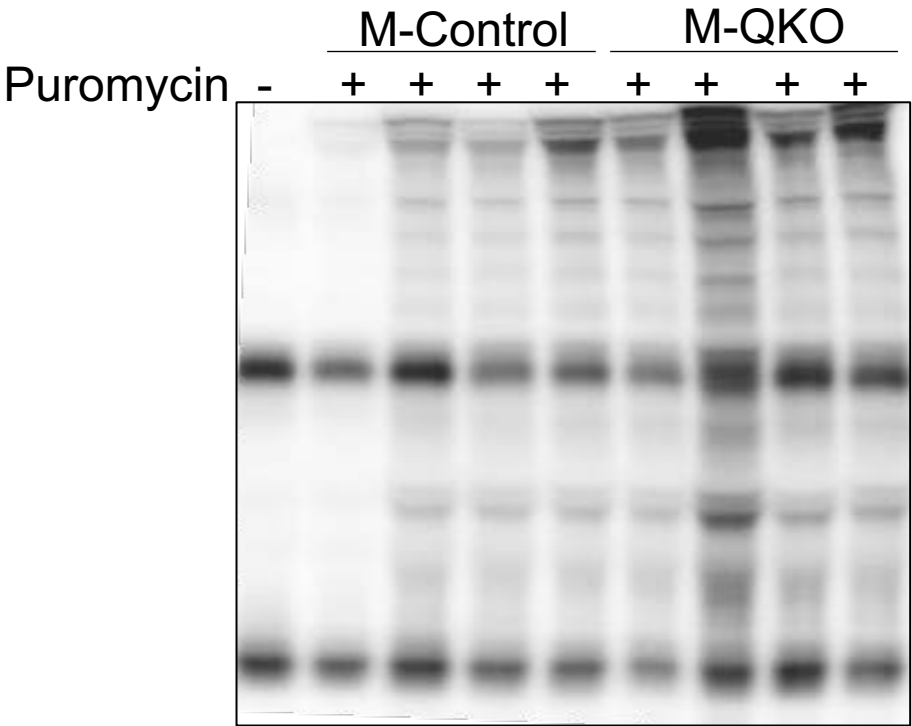

b

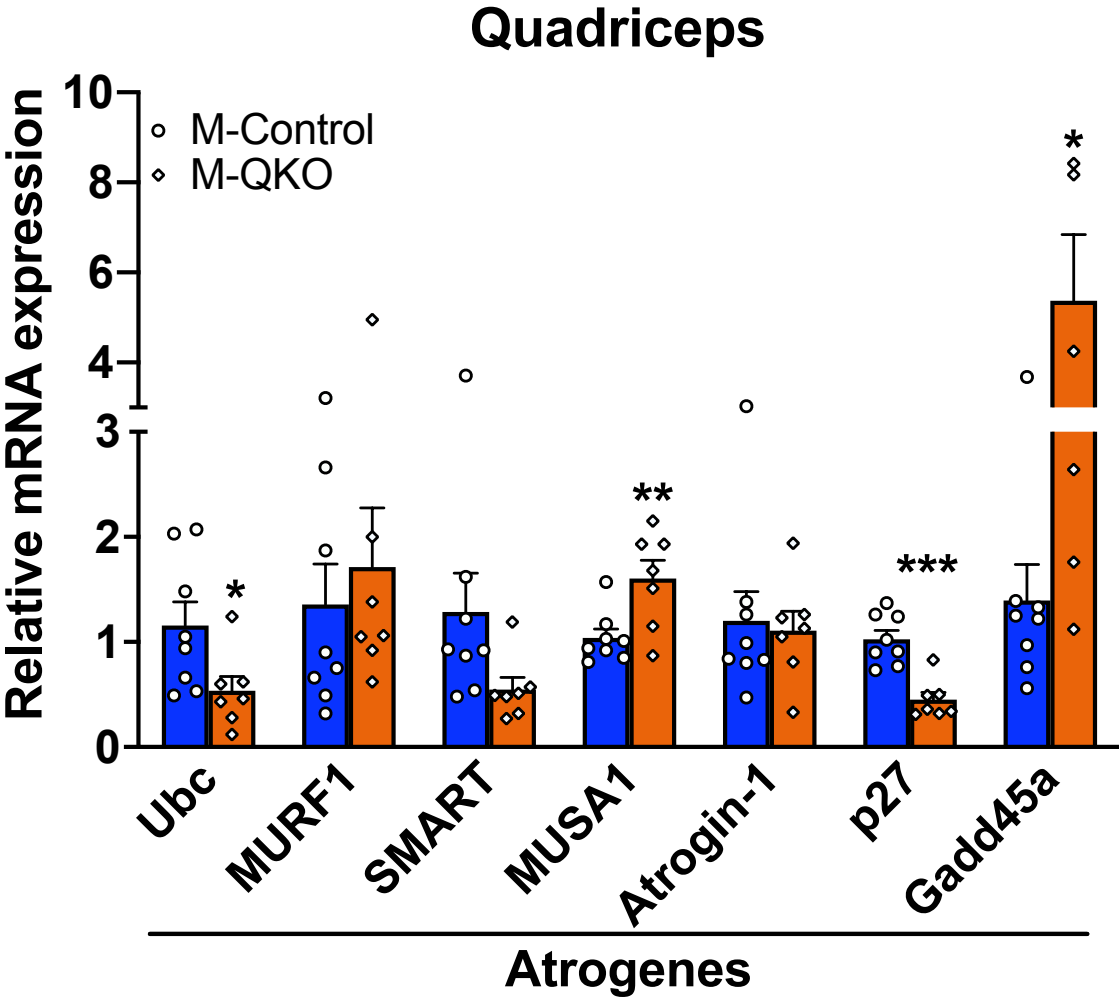

Figure S4

a

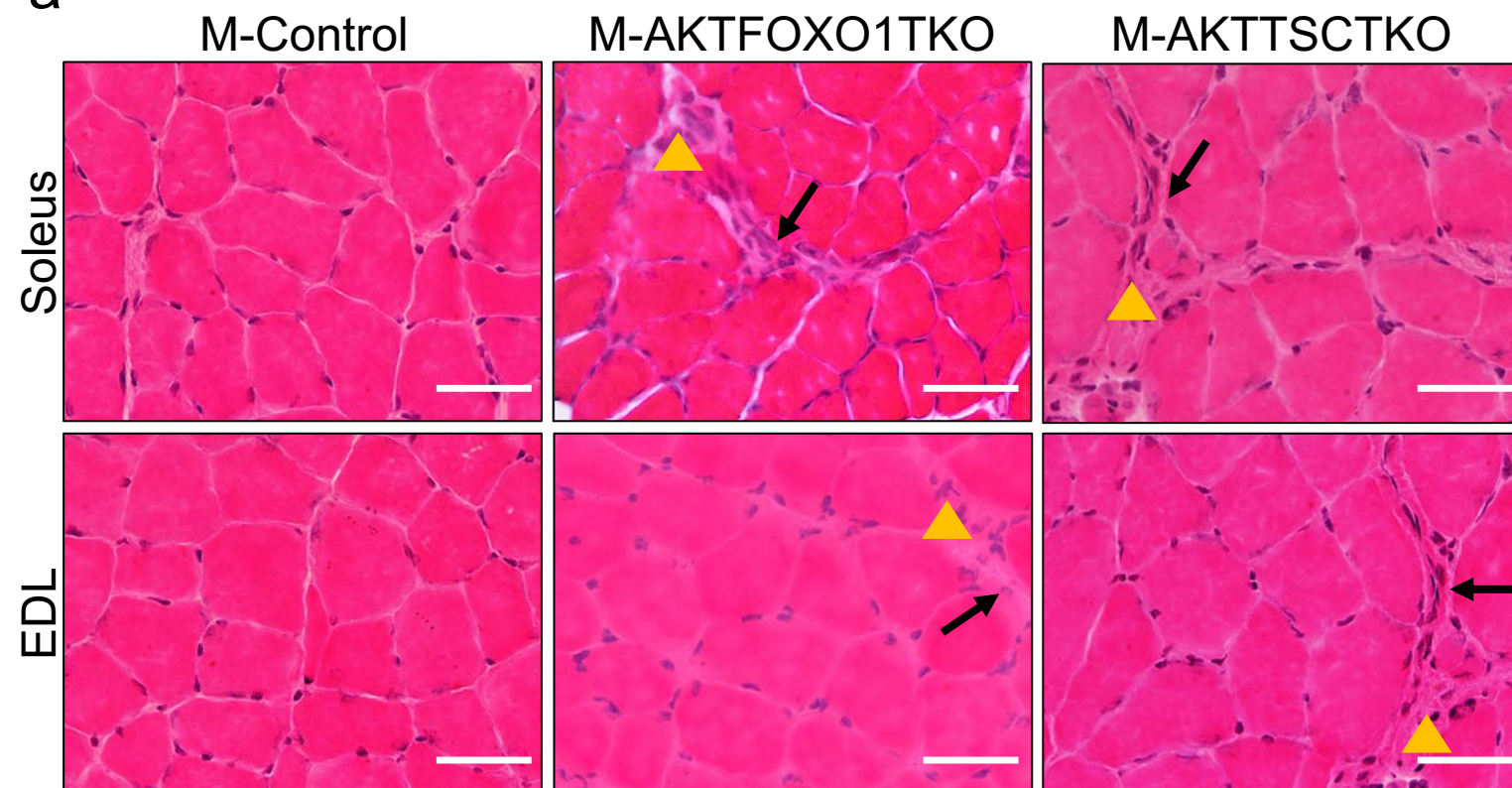

b

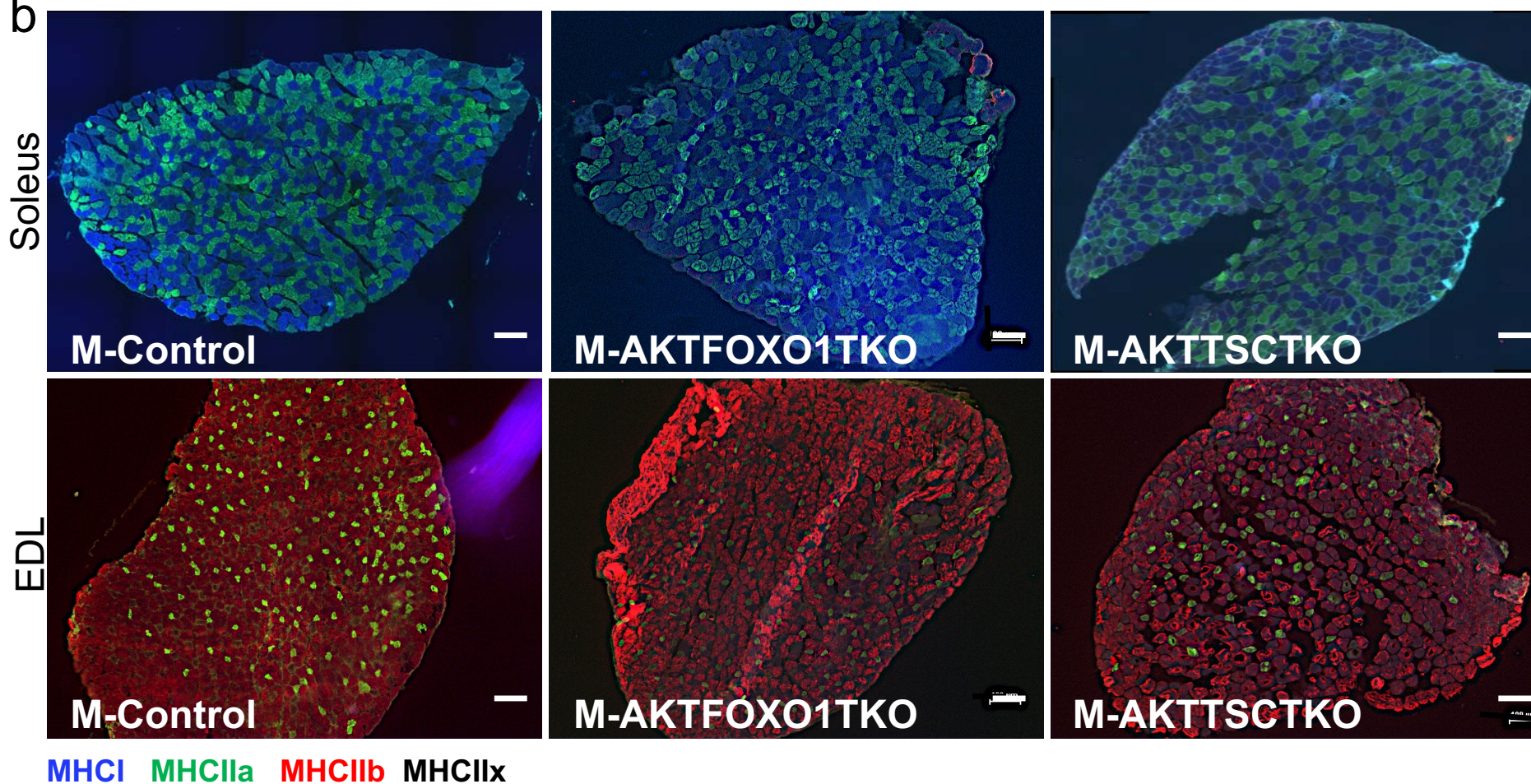

c

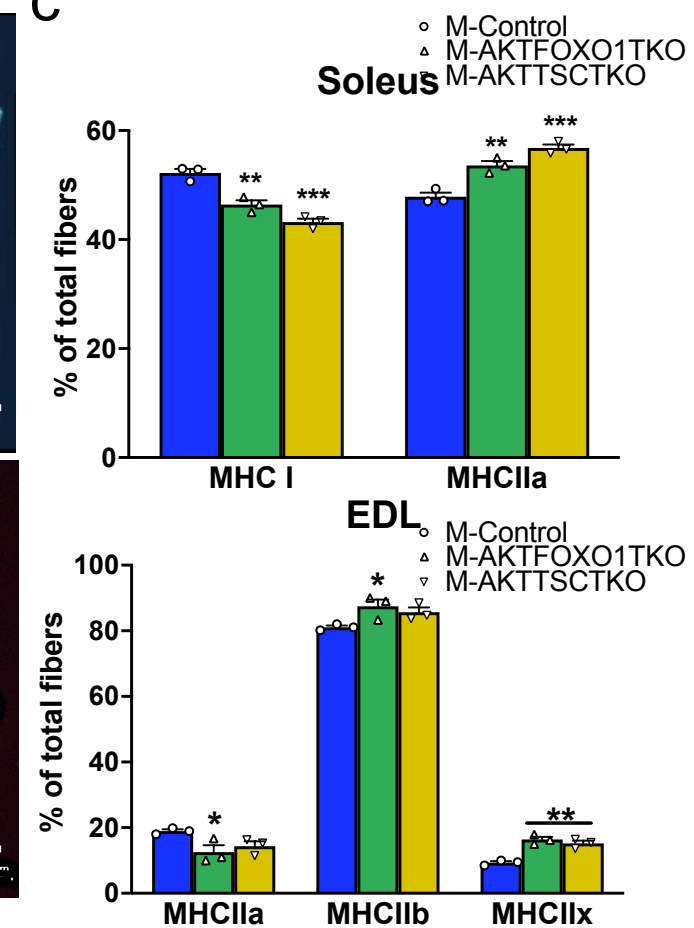

Figure S5

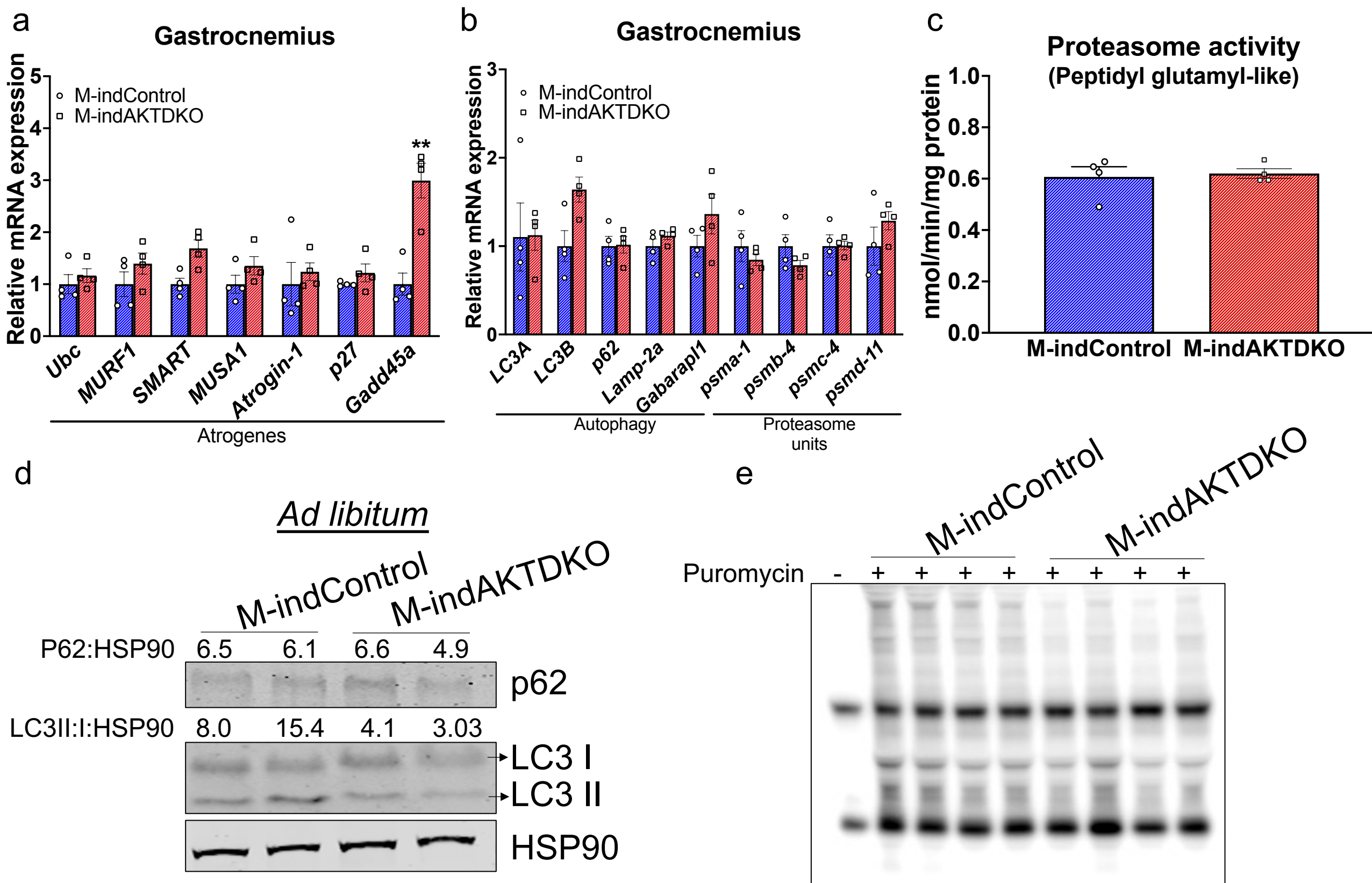

Figure S6

a

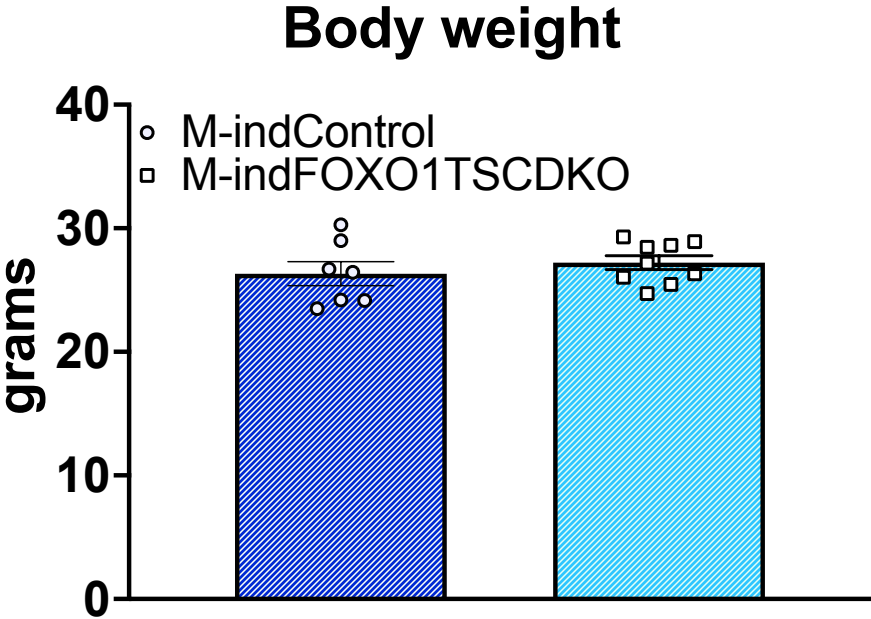

b

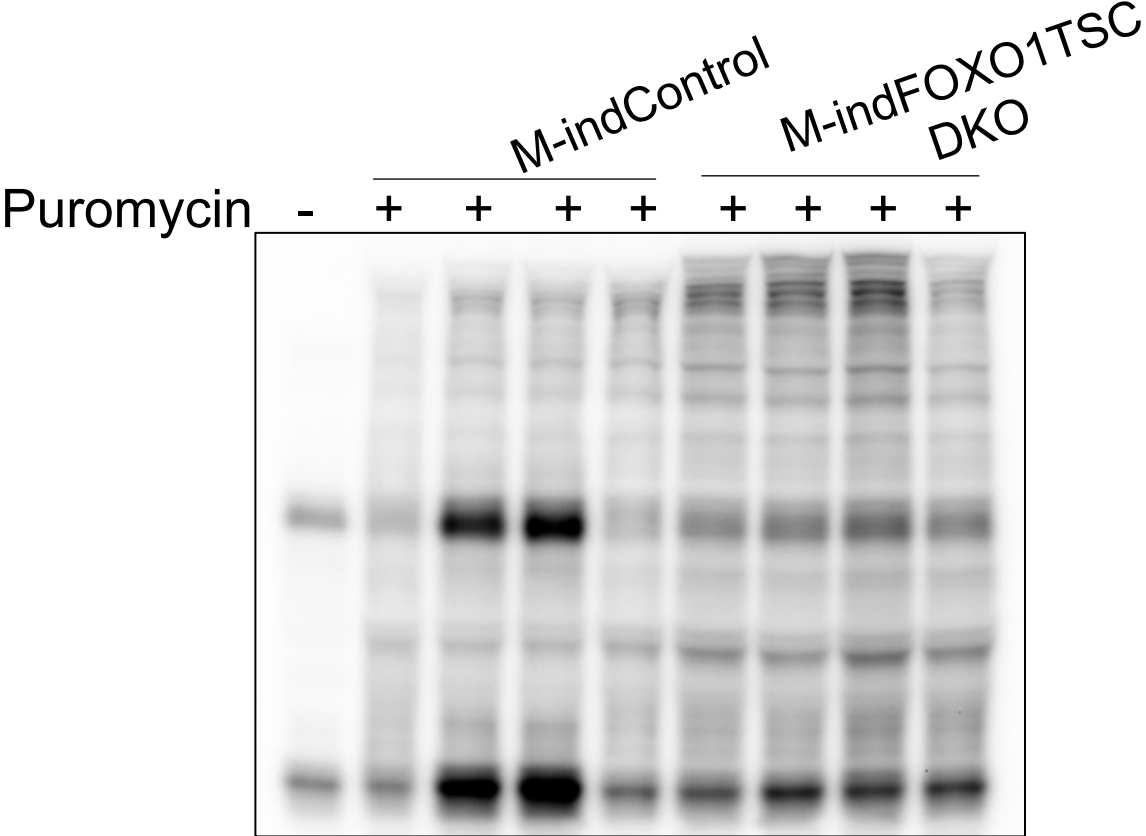

c

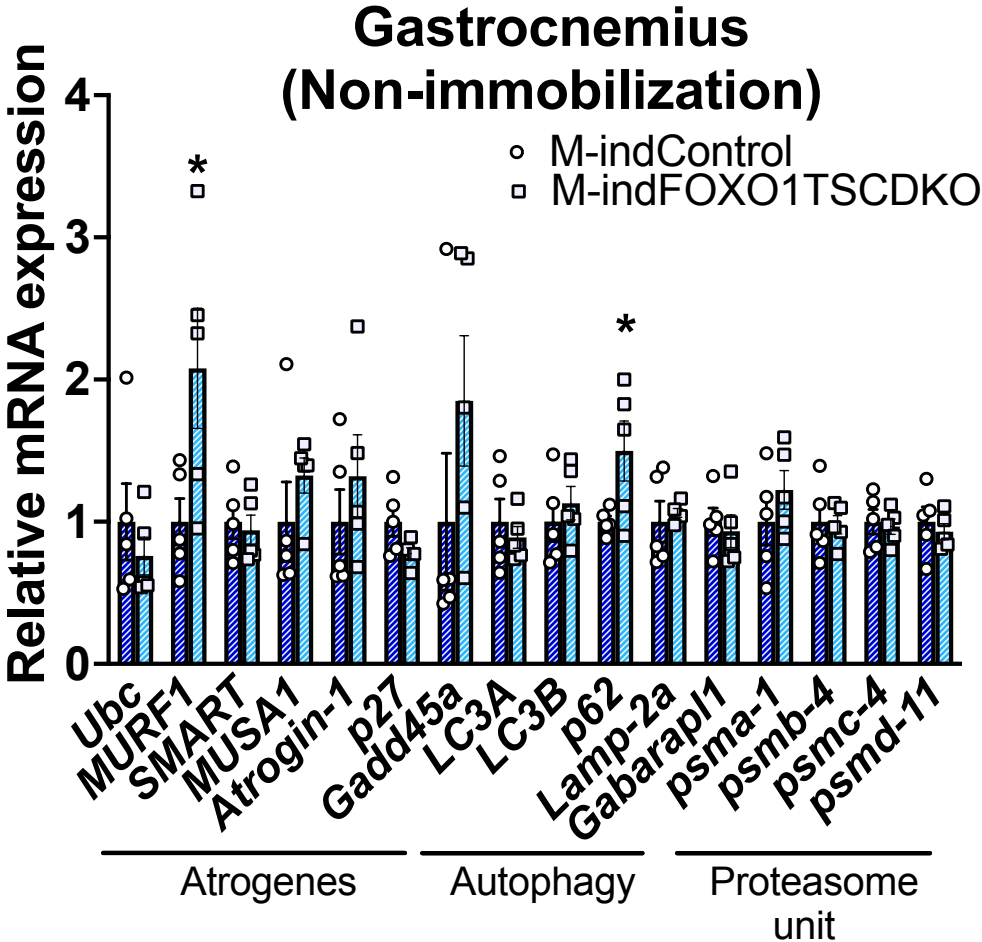

d

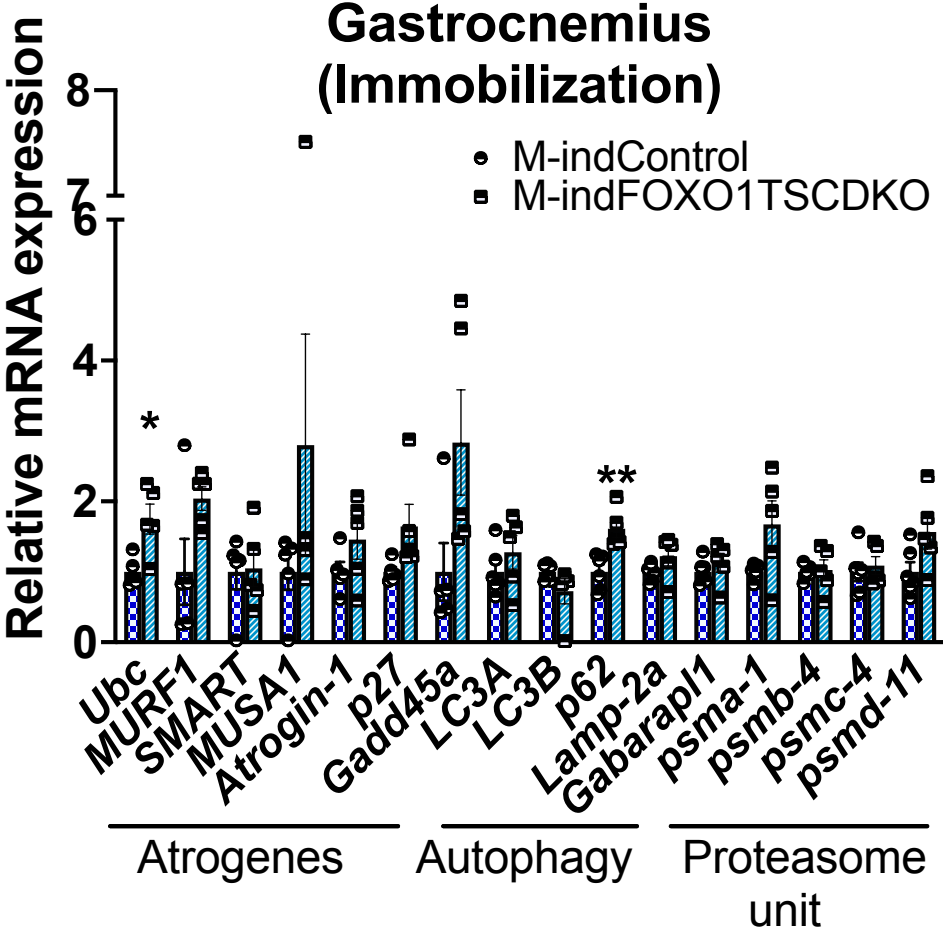

Supplement: Supplementary file 1 — Figure S1. Deletion of AKT in skeletal muscle does not affect amino acid levels or upregulate autophagy (a) Muscle weight is to body weight ratio in M‐AKTDKO muscles compared to control muscles (n = 12 for controls and n = 20 for knockouts) (b) qPCR of proteasome subunits in gastrocnemius muscle of M‐AKTDKO mice compared to controls in ad libitum state (n = 5–7) (c and d) Essential amino acid levels, BCAA and aromatic amino acid levels measured by metabolomic analysis in gastrocnemius muscles of M‐AKTDKO mice compared to M‐Control mice (n = 5) and in plasma using mass spectrometry (n = 6–7) (e) Western blots for autophagy intermediates in gastrocnemius from fed or 16 h fasted M‐AKTDKO vs M‐Control with densitometric analysis of p62 or ratio of LC3‐II/LC3‐I normalized to HSP90 (f) Relative mRNA expression of autophagy genes in gastrocnemius muscle of M‐AKTDKO mice compared to controls in ad libitum state (n = 5–12) (g) Representative blot and quantification for puromycin incorporation in gastrocnemius muscles following 16 h fasting in M‐indAKTDKO muscles (n = 4–5) (h) Representative blot for puromycin labelling in M‐AKTDKO gastrocnemius muscles compared to the control muscles refed for 1 h following 16 h fasting (n = 4) (i) Protein fractional synthesis rate in M‐AKTDKO gastrocnemius muscles compared to the control muscles using deuterium incorporation rate over 36 h. Muscles were harvested following fasting for the last 16 h (n = 4–5) (*P < 0.05, **P < 0.01, ****P < 0.0001 vs. control, data are presented as mean ± SEM). Figure S2. Inhibition of FOXO1 and activation of mTORC1 alone are not sufficient to induce muscle growth in the absence of AKT (a) Western blot of AKT2, FOXO1, phospho‐S6, S6, phospho‐4EBP1 and HSP90 in of M‐AKTTSCTKO and (b) M‐AKTFOXO1TKO gastrocnemius muscle compared to their floxed littermates. (c) Muscle weights of TA, EDL, soleus (SOL) and gastrocnemius (GAST) muscles of M‐Control and knockout mice (n = 18 for controls, n = 16 for M‐AKTTSCTKO and [file JCSM-13-495-s001.pdf]
